# Single-cell transcriptomic atlas of mouse cochlear aging

**DOI:** 10.1093/procel/pwac058

**Published:** 2022-11-11

**Authors:** Guoqiang Sun, Yandong Zheng, Xiaolong Fu, Weiqi Zhang, Jie Ren, Shuai Ma, Shuhui Sun, Xiaojuan He, Qiaoran Wang, Zhejun Ji, Fang Cheng, Kaowen Yan, Ziyi Liu, Juan Carlos Izpisua Belmonte, Jing Qu, Si Wang, Renjie Chai, Guang-Hui Liu

**Affiliations:** State Key Laboratory of Stem Cell and Reproductive Biology, Institute of Zoology, Chinese Academy of Sciences, Beijing 100101, China; University of Chinese Academy of Sciences, Beijing 100049, China; State Key Laboratory of Stem Cell and Reproductive Biology, Institute of Zoology, Chinese Academy of Sciences, Beijing 100101, China; University of Chinese Academy of Sciences, Beijing 100049, China; State Key Laboratory of Bioelectronics, Jiangsu Province High-Tech Key Laboratory for Bio-Medical Research, School of Life Sciences and Technology, Southeast University, Nanjing 211189, China; CAS Key Laboratory of Genomic and Precision Medicine, Beijing Institute of Genomics, Chinese Academy of Sciences, Beijing 100101, China; Aging Translational Medicine Center, International Center for Aging and Cancer, Beijing Municipal Geriatric Medical Research Center, Xuanwu Hospital, Capital Medical University, Beijing 100053, China; University of Chinese Academy of Sciences, Beijing 100049, China; Institute for Stem Cell and Regeneration, CAS, Beijing 100101, China; China National Center for Bioinformation, Beijing 100101, China; CAS Key Laboratory of Genomic and Precision Medicine, Beijing Institute of Genomics, Chinese Academy of Sciences, Beijing 100101, China; University of Chinese Academy of Sciences, Beijing 100049, China; Institute for Stem Cell and Regeneration, CAS, Beijing 100101, China; China National Center for Bioinformation, Beijing 100101, China; State Key Laboratory of Membrane Biology, Institute of Zoology, Chinese Academy of Sciences, Beijing 100101, China; Institute for Stem Cell and Regeneration, CAS, Beijing 100101, China; Beijing Institute for Stem Cell and Regenerative Medicine, Beijing 100101, China; The Fifth People’s Hospital of Chongqing, Chongqing 400062, China; State Key Laboratory of Membrane Biology, Institute of Zoology, Chinese Academy of Sciences, Beijing 100101, China; Institute for Stem Cell and Regeneration, CAS, Beijing 100101, China; Beijing Institute for Stem Cell and Regenerative Medicine, Beijing 100101, China; Advanced Innovation Center for Human Brain Protection, National Clinical Research Center for Geriatric Disorders, Xuanwu Hospital Capital Medical University, Beijing 100053, China; CAS Key Laboratory of Genomic and Precision Medicine, Beijing Institute of Genomics, Chinese Academy of Sciences, Beijing 100101, China; University of Chinese Academy of Sciences, Beijing 100049, China; China National Center for Bioinformation, Beijing 100101, China; State Key Laboratory of Stem Cell and Reproductive Biology, Institute of Zoology, Chinese Academy of Sciences, Beijing 100101, China; Institute for Stem Cell and Regeneration, CAS, Beijing 100101, China; Beijing Institute for Stem Cell and Regenerative Medicine, Beijing 100101, China; National Laboratory of Biomacromolecules, CAS Center for Excellence in Biomacromolecules, Institute of Biophysics, Chinese Academy of Sciences, Beijing 100101, China; University of Chinese Academy of Sciences, Beijing 100049, China; State Key Laboratory of Membrane Biology, Institute of Zoology, Chinese Academy of Sciences, Beijing 100101, China; Institute for Stem Cell and Regeneration, CAS, Beijing 100101, China; Beijing Institute for Stem Cell and Regenerative Medicine, Beijing 100101, China; Shandong Provincial Hospital and School of Laboratory Animal Science, Shandong First Medical University, Jinan 250000, China; Gene Expression Laboratory, Salk Institute for Biological Studies, La Jolla, CA 92037, USA; State Key Laboratory of Stem Cell and Reproductive Biology, Institute of Zoology, Chinese Academy of Sciences, Beijing 100101, China; University of Chinese Academy of Sciences, Beijing 100049, China; Institute for Stem Cell and Regeneration, CAS, Beijing 100101, China; Beijing Institute for Stem Cell and Regenerative Medicine, Beijing 100101, China; Advanced Innovation Center for Human Brain Protection, National Clinical Research Center for Geriatric Disorders, Xuanwu Hospital Capital Medical University, Beijing 100053, China; Aging Translational Medicine Center, International Center for Aging and Cancer, Beijing Municipal Geriatric Medical Research Center, Xuanwu Hospital, Capital Medical University, Beijing 100053, China; The Fifth People’s Hospital of Chongqing, Chongqing 400062, China; State Key Laboratory of Bioelectronics, Jiangsu Province High-Tech Key Laboratory for Bio-Medical Research, School of Life Sciences and Technology, Southeast University, Nanjing 211189, China; Institute for Stem Cell and Regeneration, CAS, Beijing 100101, China; Co-Innovation Center of Neuroregeneration, Nantong University, Nantong 226001, China; State Key Laboratory of Membrane Biology, Institute of Zoology, Chinese Academy of Sciences, Beijing 100101, China; Advanced Innovation Center for Human Brain Protection, National Clinical Research Center for Geriatric Disorders, Xuanwu Hospital Capital Medical University, Beijing 100053, China; University of Chinese Academy of Sciences, Beijing 100049, China; Institute for Stem Cell and Regeneration, CAS, Beijing 100101, China; Beijing Institute for Stem Cell and Regenerative Medicine, Beijing 100101, China

**Keywords:** single-cell transcriptomic atlas, mouse, cochlea, aging

## Abstract

Progressive functional deterioration in the cochlea is associated with age-related hearing loss (ARHL). However, the cellular and molecular basis underlying cochlear aging remains largely unknown. Here, we established a dynamic single-cell transcriptomic landscape of mouse cochlear aging, in which we characterized aging-associated transcriptomic changes in 27 different cochlear cell types across five different time points. Overall, our analysis pinpoints loss of proteostasis and elevated apoptosis as the hallmark features of cochlear aging, highlights unexpected age-related transcriptional fluctuations in intermediate cells localized in the stria vascularis (SV) and demonstrates that upregulation of endoplasmic reticulum (ER) chaperon protein HSP90AA1 mitigates ER stress-induced damages associated with aging. Our work suggests that targeting unfolded protein response pathways may help alleviate aging-related SV atrophy and hence delay the progression of ARHL.

## Introduction

Age-related hearing loss (ARHL), also known as presbycusis, is a progressive and irreversible hearing impairment caused by cochlear aging. According to epidemiologic studies, the risk of developing ARHL increases rapidly after the age of 40, with a third of people over 65 estimated to be suffering from disabling hearing loss (Eggermont, 2017; Wattamwar et al., 2017; Slade et al., 2020; Wang and Puel, 2020). Owing to hindering communication and leading to social isolation, presbycusis is often associated with depression or cognitive decline, which may in turn contribute to Alzheimer’s disease (Lin et al., 2011; Fellinger et al., 2012; Ray et al., 2018; Jafari et al., 2019; Slade et al., 2020). However, we currently have a poor understanding of the regulatory mechanisms at the cellular and molecular levels underlying age-related cochlear degeneration.

To enable hearing, sound waves are converted into vibrations in the outer and middle ear compartments, which are then converted into fluid waves by a highly specialized set of structural and functional components in the inner ear (Ni et al., 2014; Areias et al., 2016). The inner ear is a fluid-filled compartment, which is small but intricately structured. It contains the equilibrium apparatus named vestibular organ (semicircular canals, utricle, and saccule) and the hearing organ named cochlea, in which hair cells transmit auditory information to the cerebral cortex via the eighth cranial nerve (Swartz, 2008; Sanders and Gillig, 2010). The hair cells reside in the organ of Corti, a part of the cochlea where many types of supporting cells are located, and are innervated by spiral ganglion neurons. The modiolus is a spongy bone structure that forms the central axis of the cochlea and houses several different types of cells, including spiral ganglion neurons (the bipolar neurons that transmit all the auditory input to the brain), Schwann cells and satellite glial cells (Jeon et al., 2011). Another important cochlear structure, the stria vascularis (SV), is mainly composed of basal cells, intermediate cells (ICs), and marginal cells (Trowe et al., 2011; Korrapati et al., 2019), which are wrapped by the spiral ligament that contains fibrocytes that produce connective tissue proteins (Furness, 2019). In conjunction with the spiral ligament, the SV powers the endocochlear potential, the high K^+^ gradient, vital for sound sensation and audition (Bowl and Dawson, 2019; Heeringa and Koppl, 2019; Li et al., 2020b). Depending upon which cochlear region is primarily deteriorated, ARHL is divided into at least four major subtypes: sensory (loss of hair cells), neural (loss of cochlear neurons), metabolic (atrophy of SV), and cochlear conductive (changes in the conduction or resonance of the cochlear duct) (Schuknecht and Gacek, 1993; Jafari et al., 2019). However, the precise mechanisms that cause age-related degeneration in each cochlear structure remain largely unknown.

Although the structural and cellular anatomy of the cochlea has been rigorously described, a high-resolution and in-depth molecular analysis of this organ that can help us understand how age-related molecular changes cause hearing loss remains outstanding. In broadly related work, single-cell RNA sequencing (scRNA-seq) approaches have identified transcriptional signatures associated with aging and related diseases within multiple organs and heterogeneous tissues (Angelidis et al., 2019; He et al., 2020; Ma et al., 2020; Tabula Muris, 2020; Wang et al., 2020a, 2021a; Zhang et al., 2020, 2022; Li et al., 2021; Zou et al., 2021; Cai et al., 2022; Leng and Pawelec, 2022; Zhou et al., 2022). However, scRNA-seq has not yet been applied to systematically map and molecularly profile the structurally and functionally distinct cochlear compartments, or potential age-dependent changes.

In this study, we established a comprehensive single-cell transcriptomic atlas of mouse cochlear aging at high-temporal resolution. In the SV, we found that ICs showed pronounced transcriptional alterations during aging, among which elevation of unfolded protein response (UPR) and apoptosis were the most profound. Notably, we discovered that the chaperon HSP90AA1 was elevated during aging, and the activation of endogenous HSP90AA1 alleviated ER stress-induced damages in SV cells, suggesting a compensatory mechanism that may help to prevent SV from aging-associated atrophy. Collectively, our study constitutes a rich resource for identification of cell type-specific molecular alterations along the time course of cochlear aging, and as such, will enable the discovery of cochlear aging-associated biomarkers, and help identify cellular and molecular programs that can be targeted therapeutically in presbycusis.

## Results

### Aging-associated cochlear disorganization and functional decline in mouse

**Figure 1.**
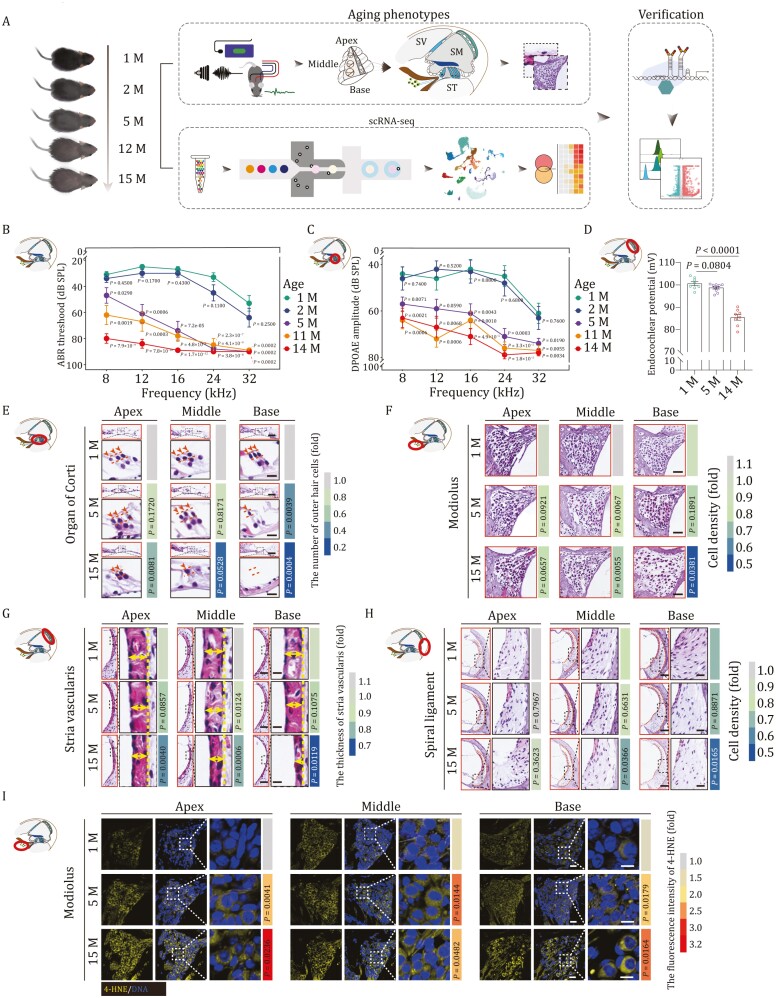
**Aging-related phenotypes of mouse cochlea.** (A) Diagram showing the procedure of aging phenotypical analysis, single cell RNA sequencing (scRNA-seq), and subsequent verification of molecular mechanism. Month, M; Scala Vestibuli, SV; Scala Media, SM; Scala Tympani, ST. (B and C) Line charts showing ABR (B) and DPOAE (C) thresholds of 1-, 2-, 5-, 11-, and 14-month-old mice in response to different frequencies. The ABR or DPAOE thresholds of 2-, 5-, 11-, and 14-month-old mice were compared with those of 1-month-old mice. The quantitative data are presented as the mean ± SEMs (*n* = 10 mice). Two-tailed Student’s *t*-test *P*-values were indicated. Month, M. (D) A recording of the endocochlear potential of 1, 5, and 14 months old mice (1 M, *n* = 8 mice; 5 M, *n* = 10 mice; 14 M, *n* = 8 mice). Data were shown as the mean ± SEMs. Two-tailed Student’s *t*-test *P*-values were indicated. Month, M. (E) H&E-staining of outer hair cells in the apical, middle, and basal turns of cochleae from 1-, 5-, and 15-month-old mice. Scale bars, 40 and 10 μm (zoomed-in images). The cell number was counted and quantified. The number of cells is quantified as fold changes relative to that of apical turn in 1-month-old cochlea (*n* = 5 mice). Two-tailed Student’s *t*-test *P*-values were indicated. Month, M. (F) H&E staining of modiolus in the apical, middle, and basal turns from 1-, 5-, and 15-month-old mouse cochleae. Scale bars, 40 μm. The cell density is quantified as fold changes relative to that of apical turn in 1-month-old cochlea (*n* = 5 mice). Two-tailed Student’s *t*-test *P*-values were indicated. (G) H&E staining of stria vascularis in the apical, middle, and basal turns of cochleae from 1-, 5-, and 15-month-old mice. Scale bars, 40 μm and 10 μm (zoomed-in images). The thickness of stria vascularis was counted and quantified as fold changes relative to that of apical turn in 1-month-old cochlea (*n* = 5 mice). Two-tailed Student’s *t*-test *P*-values were indicated. (H) H&E staining of spiral ligament in the apical, middle, and basal turns of cochleae from 1-, 5-, and 15-month-old mice. Scale bars, 80 and 20 μm (zoomed-in images). The apical, middle, and basal spiral ligament cell density were counted and quantified as fold changes relative to that of apical turn in 1-month-old cochlea (*n* = 5 mice). Two-tailed Student’s *t*-test *P*-values were indicated. (I) 4-HNE immunofluorescence staining showed elevated intensity of 4-HNE in 5- or 15-month-old mice compared with that in 1-month-old mice. Scale bars, 40 and 10 μm (zoomed-in images). The relative intensity is quantified as fold changes relative to that of apical turn in 1-month-old cochlea (*n* = 5 mice). Two-tailed Student’s *t*-test *P*-values were indicated.

To uncover the cellular and molecular mechanisms of cochlear aging, we used C57BL/6J mice, a widely used rodent model of ARHL (Willott, 2009; Kane et al., 2012; Oike et al., 2021). To establish a timeline for assessing phenotypic changes occurring during cochlear aging in C57BL/6J mice, we first examined their hearing abilities at different ages by auditory brainstem responses (ABRs) and distortion product otoacoustic emissions (DPOAEs) (Fig. 1A–C). Consistent with previous studies, the frequency-specific ABR and DPOAE thresholds increased with age (Fig. 1B and 1C), suggesting that severe hearing loss has already appeared around the age of 5 months (Hequembourg and Liberman, 2001; Kane et al., 2012; Mellado Lagarde et al., 2014; Zhang et al., 2015). In addition, the endocochlear potential also showed a progressive decrease with age, and by the age of 14 months it had reached a marked reduction (Fig. 1D), reflecting loss of voltage potential in the scala media, which further impaired signaling transduction. Next, to identify structural alterations underlying aging-associated compromised hearing, we collected cochlear tissues from mice aged 1, 5, and 15 months for histological analysis. In hematoxylin and eosin (H&E)-stained sections, the overall structure of the cochlea in the middle-aged (5-month-old) and old (15-month-old) mice appeared largely comparable to that in young counterparts (1-month-old) (Fig. 1E–H). However, when we more closely compared different anatomical regions, including the organ of Corti, the modiolus, the SV, and the spiral ligament, we found that aging differentially affects these regions. Specifically, in the organ of Corti, outer hair cells (OHCs) and inner hair cells (IHCs) were markedly lost, especially in the basal turn (Figs. 1E and S1A–C), in agreement with the known and prominent loss of high-frequency hearing at an advanced age (Mianne et al., 2016; Fu et al., 2018; Heeringa and Koppl, 2019; Keithley, 2020). Similarly, in 15-month-old mice, we found that overall cell density and the density of Tuj1-positive pan-neurons in the modiolus were reduced, particularly in the basal turn of the cochlea (Figs. 1F, S1D and S1E). Although the cell density in the SV in aged mice was comparable to that in the young cochlea, the regional thickness was decreased throughout the entire cochlear region (Figs. 1G, S1F and S1G), consistent with aging-associated SV atrophy known to underlie imbalanced endocochlear potential (Fig. 1D). In the spiral ligament, the thickness did not appear to be impacted by aging. However, the cell density of both middle and basal turns was lower than it was in the young cochlea (Figs. 1H and S1H). In addition, a set of age-related damages accumulated with advanced age. For instance, in line with prior reports of elevated inflammation in the aged cochlea (Watson et al., 2017; Bae et al., 2021), we found a massive accumulation of infiltrated neutrophils in spiral ligament of the aged cochlea (Fig. S1J), particularly in the apical and middle turns. Furthermore, levels of the lipid oxidation marker 4-hydroxynonenoic acid (4-HNE) were increased throughout the aged modiolus (Figs. 1I and S1I), reflecting enhanced oxidative damage in cochlea with age. Altogether, these results demonstrated that the cochlea underwent both structural and functional degeneration during physiological aging.

### Comprehensive cellular and molecular taxonomy of cochlea based on scRNA-seq

**Figure 2.**
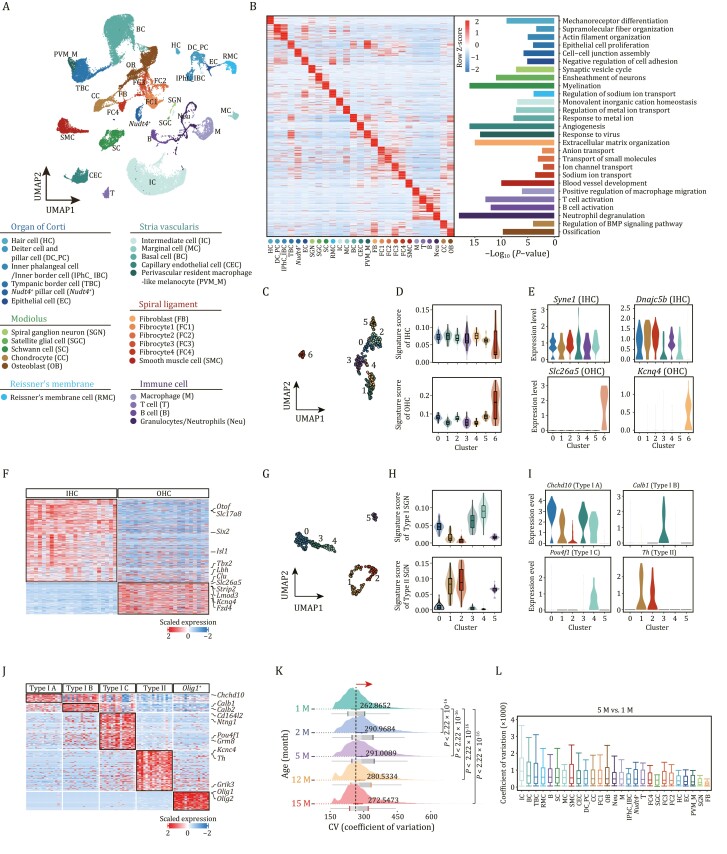
**Establishment of single-cell transcriptome landscape of mouse cochlea.** (A) Top, UMAP plot showing the distribution of different cell types in cochlea. Bottom, the annotation of different cell types. HC, Hair cell; DC_PC, Deiter cell and pillar cell; IPhC_IBC, Inner phalangeal cell/inner border cell; TBC, Tympanic border cell; *Nudt4*^+^, *Nudt4*^+^ pillar cell; EC, Epithelial cell; SGN, Spiral ganglion neuron; SGC, Satellite glial cell; SC, Schwann cell; RMC, cells in Reissner’s membrane; IC, Intermediate cell; MC, Marginal cell; BC, Basal cell; CEC, Capillary endothelial cell; SMC, Smooth muscle cell; PVM_M, Perivascular resident macrophage-like melanocyte; FB, fibroblast; FC1, Fibrocyte 1; FC2, Fibrocyte 2; FC3, Fibrocyte 3; FC4, Fibrocyte 4; T, T cell; B, B cell; M, Macrophage; Neu, Granulocyte/neutrophil; CC, Chondrocyte; OB, Osteoblast. (B) Left, heatmap showing row z-score expression signatures of top 50 cell-type-specific genes. Right, representative Gene Ontology (GO) terms for marker genes. (C) UMAP plot showing the distribution of subclusters of HCs. (D) Violin and box plots showing the gene set scores of IHC (top) or OHC (bottom) signature genes in different subpopulations of HCs. Boxes show the medians and the quartile ranges (25%–75%), while the lengths of the whiskers represent 1.5× the IQR. (E) Violin plots showing the expression levels of select marker genes that are differentially expressed in IHCs and OHCs. *Syne1* and *Dnajc5b* mark the IHCs. *Slc26a5* and *Kcnq4* mark the OHCs. (F) Heatmap showing the gene expression signatures of IHCs and OHCs. (G) UMAP plot showing the distribution of subclusters of SGNs. (H) Violin and box plots showing the gene set score of type I (top) or II (bottom) signature genes in different subpopulations of SGN. Boxes show the medians and the quartile ranges (25%–75%), while the lengths of the whiskers represent 1.5× the IQR. (I) Violin plots showing the expression levels of selected marker genes that are differentially expressed in types I and II SGN. (J) Heatmap showing the gene expression signatures of SGN subtypes. (K) Ridge plot showing the shift of CV of cochlear cells with age. *P* values by Wilcoxon test are indicated. (L) Box plot showing the CV of each cell type at 5-month-old compared to that of 1-month-old. Box shows the median and the quartile range (25%–75%) and the length of whiskers represents 1.5× the IQR.

To unveil the gene expression dynamics of cochlear aging at temporal resolution, we constructed a high-throughput scRNA-seq atlas of the mouse cochlea spanning five time points: 1, 2, 5, 12, and 15 months of age (Figs. 1A, 2A, and S2E). After strict quality control (see Methods), we obtained 45,972 single-cell transcriptomes for downstream analysis (Fig. S2A–D) and applied uniform manifold approximation and projection (UMAP) analysis to resolve the cell type distribution for each time point (Figs. 2A and S2E). Since we did not find significant gender differences between female and male C57BL/6J mice in the transcriptomic profiling, we presented the data by combining the female and male samples of the same age (Fig. S2F and S2G). Through gene-expression profile analysis and based on well-defined cell type-specific markers (Figs. 2A, S2E, S3A and S3B; Table S1), we identified 27 major cell types that were distributed across six groups, including cells localizing in and around the organ of Corti, and cells localizing in modiolus, Reissner’s membrane, SV and spiral ligament, as well as different types of immune cells (Fig. 2A). Cells localizing in and around the organ of Corti included hair cell (HC, *Pou4f3*^*+*^, *Pcp4*^*+*^), Deiter cell and pillar cell (DC_PC, *Fbxo2*^*+*^, *Skp1a*^*+*^), *Nudt4*^*+*^ pillar cell (*Nudt4*^*+*^), and inner phalangeal cell/inner border cell (IPhC_IBC, *Slc1a3*^*+*^, *S100a6*^*+*^) (Figs. 2A, 2B and S3C–H) (Nelson et al., 2007; Oshima et al., 2010; Wan et al., 2014; Burns et al., 2015; Waldhaus et al., 2015; Zhou and Hu, 2015; Hoa et al., 2020; Chen et al., 2021). Cells in the modiolus comprised five major cell types: spiral ganglion neuron (SGN, *Nefh*^*+*^, and *Snap25*^*+*^), satellite glial cell (SGC, *Mog*^+^, and *Tubb4a*^+^), Schwann cell (SC, *Mpz*^*+*^, and *Pmp22*^*+*^), chondrocyte (CC, *Prg4*^*+*^, and *Slc26a2*^+^) (Haila et al., 2001; Schmidt et al., 2004; Park et al., 2014; Abubacker et al., 2016; Milon et al., 2021), and osteoblast (OB, *Dio2*^+^, and *Runx2*^+^) (Samee et al., 2008; Wei et al., 2015) (Figs. 2A and S3A). SV was constituted of four major cell types: IC (*Kcnj10*^*+*^, and *Kcnj13*^*+*^), marginal cell (MC, *Kcnq1*^*+*^, and *Kcne1*^*+*^), basal cell (BC, *Tjp1*^high^), and capillary endothelial cell (CEC, *Ly6c1*^*+*^, and *Vwf*^*+*^) (Figs. 2A and S3A) (Au-Yeung et al., 2004; Wangemann et al., 2004; Jabba et al., 2006; Ohlemiller et al., 2009; Yang et al., 2009; Kim et al., 2013; Vanlandewijck et al., 2018; Korrapati et al., 2019; Brulois et al., 2020). Cells in the spiral ligament mainly included four fibrocyte subtypes, fibroblast, and smooth muscle cell (SMC). Other cell types included cell in Reissner’s membrane (RMC, *Vmo1*^*+*^, and *Slc26a7*^*+*^) (Figs. 2A and S3A) (Kim et al., 2014; Barsh et al., 2018), as well as various immune cell types (Figs. 2A and S3A). Through functional enrichment analysis of the top 50 cell type-specific marker genes, we mapped the unique function of each cell type (Fig. 2B). For example, mechanoreceptor differentiation mapped to HCs, synaptic vesicle cycle to SGNs, monovalent inorganic cation homeostasis to ICs, and angiogenesis to CECs, etc.

Furthermore, our detailed analysis also identified HC and SGN subpopulations based on their canonical marker genes and unique functions (Figs. 2C–J, S3I and S3J). For example, IHCs  and OHCs were distinguished by the expression scores for a combination of highly expressed signature genes for either cell type, with the former expressing classical marker genes including *Syne1* and *Dnajc5b*, and the latter expressing *Slc26a5* and *Kcnq4* (Li et al., 2018). Five SGN subtypes were identified based on their unique gene expression signatures, namely, type IA (*Chchd10*^+^), type IB (*Calb*^+^), type IC (*Pou4f1*^+^), type II (*Th*^+^), and an *oligo1*^+^ SGN subtype (Li et al., 2018; Petitpre et al., 2018; Shrestha et al., 2018; Sun et al., 2018). Collectively, we established a comprehensive cellular and molecular taxonomy of the adult mouse cochlea, serving as a foundation for age-dependent analysis.

### Global transcriptional changes during mouse cochlear aging

To decipher aging-related transcriptional perturbations in the mouse cochlea, we first performed an overall coefficient of variation (CV) analysis. We found that the CV increased during aging, as manifested by elevated CV values at 2-, 5-, 12-, and 15-month-old compared with that of 1-month-old, and peaked at 5-month-old (Fig. 2K). When we compared the CV across all cochlear cells between 5-month-old and 1-month-old, we discovered that ICs and basal cells located in SV, tympanic border cells (TBCs) located under the basilar membrane, and cells in Reissner’s membrane exhibited higher CV (Fig. 2L). For other pairwise comparisons across ages (2 M vs. 1 M, 12 M vs. 1 M, 15 M vs. 1 M), ICs, basal cells, TBCs, marginal cells, and Schwann cells ranked as the top five cell types with higher CV (Fig. S4A). Next, we retrieved genes for which expression correlated positively with transcriptional fluctuations in ICs harboring the highest CV between 5- and 1-month-old (Fig. 2L), and found several genes known to be associated with age-related cellular dyshomeostasis or hearing loss (Fig. S4B and S4C). For instance, *Smad5*, a known effector downstream of the TGF-β signaling pathway, is reportedly correlated with fibrogenesis and inflammation in the cochlea (Bas et al., 2020). Altogether, these analyses enabled us to capture cumulative and prominent transcriptomic heterogeneity characteristics of cochlear aging.

### Temporal-specific transcriptomic signatures during cochlear aging by pairwise differential expression analysis

**Figure 3.**
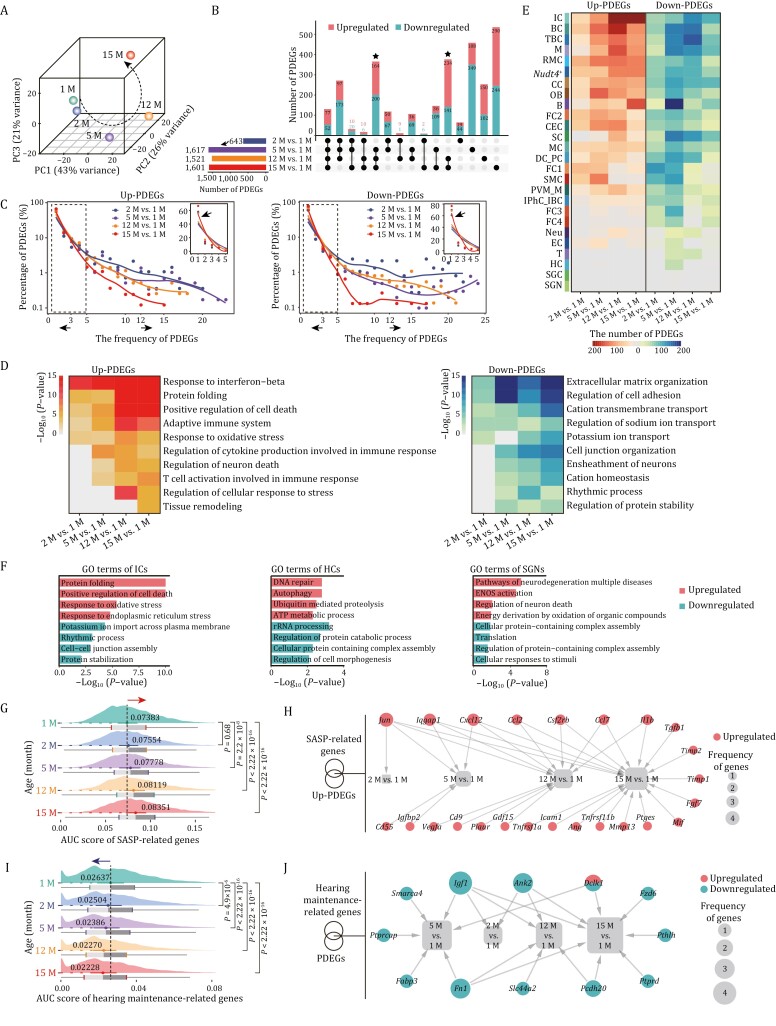
**Pairwise differential expression analysis reveals cell type-specific temporal signatures during cochlear aging.** (A) Principal component analysis (PCA) of cochlear single-cell transcriptome data from each age group. (B) Upset plot showing the numbers of age-unique and shared PDEGs for pairwise comparisons between different age groups. (C) The smooth line plots showing the expression patterns of the frequency and percentage of the upregulated (left) and downregulated (right) PDEGs between 2, 5, 12, 15 months old and 1 month old, individually. Insets show the zoomed-in view of the region highlighted by a dashed line to the left. The arrows pointing to the left represent the PDEGs with a frequency less than 3, and the arrows to the right represent the PDEGs with a frequency more than 12. (D) Representative GO terms of upregulated PDEGs (left) and downregulated PDEGs (right) across four pairwise comparisons between different age groups. (E) Heatmap showing the number of upregulated (left) and downregulated PDEGs (right) of each cell types between 2, 5, 12, 15 months old and 1 month old, respectively. (F) Representative GO terms of total PDEGs in ICs (left), HCs (middle) and SGNs (right). (G) Ridge plot showing the AUC score of SASP-related genes in cochlear cells from 1-, 2-, 5-, 12-, and 15-month-old mice. (H) Network plot showing the upregulated PDEGs overlapped with genes from SASP gene set. The node size indicates the frequency of PDEGs appeared across four pairwise comparisons. (I) Ridge plot showing the AUC score of hearing maintenance-related genes in cochlear cells from 1-, 2-, 5-, 12-, and 15-month-old mice. (J) Network plot showing the PDEGs overlapped with hearing maintenance-related genes. The node size indicates the frequency of PDEGs appeared across four pairwise comparisons.

As samples were segregated transcriptionally by age (Fig. 3A), we next sought to resolve the temporal resolution of onset and the rate of aging as manifested by age-associated gene expression alterations. Through analyzing pairwise differentially expressed genes (PDEGs) between 1-month-old mice and mice of other ages, we discovered that transcriptional changes (a total of 643 PDEGs) were already present in the cochlea of the 2-month-old mice (Fig. 3B). In addition, around one-fifth (129) of those PDEGs were shared by the other three pairwise comparisons at later stages, suggesting that the age-related gene expression changes in mice are poised in young adults, which may facilitate functional decay in later life (Fig. 3B and Table S2). A sharp increase in the number of PDEGs was observed at the age of 5-month, with relatively moderate changes occurring in 12-month and 15-month-old mice (Fig. 3B and Table S2). In addition to age-specific gene expression changes, many PDEGs overlapped at the ages of 5-, 12-, and 15-month (Fig. 3B and Table S2), suggesting that aging-associated transcriptional characteristics were largely established by 5 months of age. Taken together, the cochlear aging gene expression dynamics indicates a progressive hearing loss with advanced age, consistent with the reported phenotypes in C57BL/6J mice (Kane et al., 2012; Zhang et al., 2013).

Next, through analysis of the number and frequency (chances appearing in each cell type) of PDEGs, we observed a panel of PDEGs with high frequency at the early stage (2 M vs. 1 M), while a larger number of PDEGs emerged with low frequency at later stage (15 M vs. 1 M) (Figs. 3C, S5A–D and Table S2). This pattern implies that age-associated gene expression alterations transit from a relatively homogeneous state to a more heterogenous and cell type-specific state. Accordingly, most genes with high frequency (≥ 12) were mainly related to transcription (Upregulated: *Tceb2* and *Wbp5*; downregulated: *Elob* and *Cebpd*) and translation (Upregulated: *Rpl13a*; downregulated: *Nop53* and *Rack1*) (Fig. S5A and S5B). In contrast, upregulated low frequency (≤ 3) genes were related to positive regulation of cell death, chaperone-mediated protein folding, response to oxidative stress, and immune response (Fig. S5C). The downregulated genes with low frequency (≤ 3) were associated with extracellular matrix organization, regulation of cell adhesion, cation transmembrane transport, and ensheathment of neurons (Fig. S5D). The overall transcriptional chaos between pairwise comparisons suggests that aging PDEGs poised early in life were associated with common regulatory machinery, whereas those emerging later were involved in diverse and complicated transcriptional disturbance underlying age-related damages.

Furthermore, functional enrichment analysis and transcriptional network analysis of PDEGs from four pairwise comparisons uncovered similar yet asynchronous characteristics during cochlear aging (Figs. 3D, S6A, S6B, and S7A–C). For example, upregulated PDEGs related to response to interferon-beta and protein folding were shared by four pairwise comparisons, regulation of neuron death and regulation of cellular response to stress were shared by the later three or two pairwise comparisons, whereas genes associated with tissue remodeling was unique for 15 M vs. 1 M (Fig. 3D). To further dissect cell type-specific changes underlying the onset and rate of cochlear aging, we attributed PDEGs to each cell type (Fig. 3E). Consistent with the previous observations, the most cell types showed remarkable responses to aging at the 5-month-old (Fig. 3E). Among these, the top three cell types harboring the most upregulated and downregulated PDEGs between 5- and 1-month were the basal cell, B cell and TBC, while the IC, basal cell, and TBC showed the most aging PDEGs at 12-month (Fig. 3E). Intriguingly, the largest total number of PDEGs was found in the ICs, particularly upregulated PDEGs persisted to the month 15, and manifested by enhanced protein folding and regulation of cell death, as well as dampened rhythmic process and cell–cell junction assembly (Fig. 3E and 3F), suggesting progressive and gradual age-related loss of function in ICs. Although HCs and SGNs, two crucial functional cell types in the cochlea (Fu et al., 2018; Shrestha et al., 2018; Sun et al., 2018; Wu et al., 2020), did not exhibit marked transcriptional alterations as manifested by the numbers of PDEGs, we still noticed that the molecular alterations underlying previously observed phenotypes accumulate with age (Figs. 1E, 1F and 3F). For example, upregulated PDEGs associated with DNA repair and neuronal cell death were observed in HCs and SGNs, respectively (Fig. 3F), suggesting presence of age-accumulated impairments in these cells, which may contribute to functional decay during cochlear aging.

Next, we performed comparative analysis of newly identified age-related PDEGs and genes annotated in the Aging Atlas (Table S3) (Aging Atlas, 2021), and identified 66 overlapping aging-high-risk genes whose expression changes were shared across the four pairwise comparisons, and 48 genes with age-specific expression changes (7, 13, 12, 16 genes for four comparisons from consecutive time points) (Fig. S7D). Among the genes with shared expression changes were canonical aging markers including *Cdkn1a*, *Lmna*, *Gadd45b*, and *Gadd45g* (Magimaidas et al., 2016; Brito et al., 2020), heat shock protein-coding genes including *Hspa1b*, *Hsp90aa1*, and *Hspa8*, antioxidant protein-coding genes including *Gpx4* and *Prdx1*, and genes encoding chemokines including *Ccl19*, *Cxcl12*, *Ccl2*, and *Ccl7*, as well as gene encoding secretory factor *S100b* (Fig. S7D). Given that the senescence-associated secretory phenotype (SASP) found in senescent cells is believed to elicit chronic inflammation and contribute to organ aging (Simon et al., 2019; Birch and Gil, 2020), we next compared SASP gene set scores of all cochlear cells of different ages (Table S3). Throughout the time points, we noticed a gradual increase in gene set scores (Fig. 3G). Joint analysis of SASP-related genes and upregulated PDEGs revealed core genes including *Cxcl12*, *Ccl2*, and *Ccl7* (Fig. 3H), that likely contribute to the elevated inflammation responses in aged cochlea. Furthermore, we also calculated gene set scores for annotated hotspot genes from hearing maintenance gene set (Table S3) (Hoffmann et al., 2016; Morgan et al., 2019; Ingham et al., 2020). As expected, the scores declined with age along the successive time points (Fig. 3I), and most of the overlapping genes between PDEGs and hearing maintenance-related genes were decreased (Fig. 3J), further implying a progressive hearing loss with age.

Collectively, our findings portray the kinetics of age-dependent PDEGs underlying progressive hearing loss. Specifically, a series of accumulated responses to age-related stressors, including chronic inflammation, dysregulated protein folding, and oxidative stress along with apoptosis served as the major transcriptional features during cochlear aging.

### Cell type-specific age-dependent dynamic gene signatures in mouse cochlea

**Figure 4.**
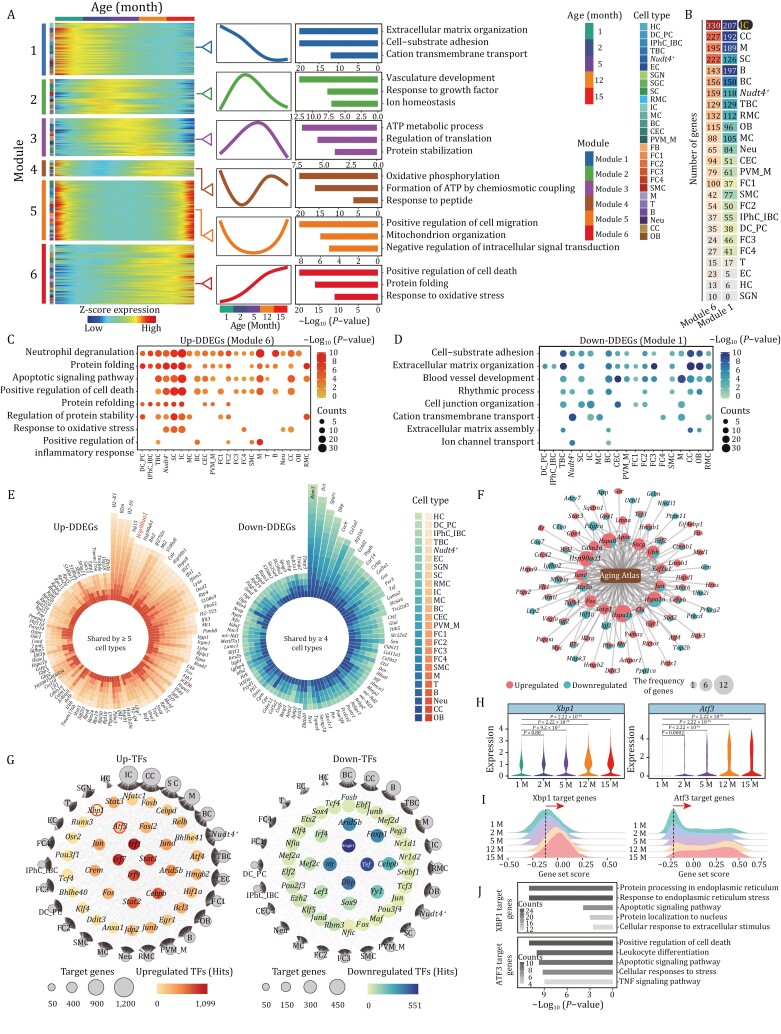
**Dynamic differential expression analysis uncovers transcriptional signatures during cochlear aging.** (A) Heatmaps showing dynamic DEGs (DDEGs) with six different expression patterns. The corresponding gene expression trajectories and representative GO terms are shown in the middle and right panels. (B) Heatmap showing the number of upregulated DDEGs (module 6) and downregulated DDEGs (module1) in each cell type. (C and D) Representative GO terms of upregulated DDEGs (module 6) (C) and downregulated DDEGs (module 1) (D) in the different cochlear cell types. Count indicates gene number. (E) Plots showing the upregulated DDEGs (left) shared by at least five cell types and downregulated DDEGs (right) shared by at least four cell types. (F) Network plot showing the upregulated and downregulated DDEGs overlapped with genes annotated in Aging Atlas database. The node size indicates the frequency of DDEGs appeared across different cell types. (G) Network visualization of upregulated (left) and downregulated (right) core regulatory transcription factors (TFs) across all cell types during cochlear aging. Outer nodes represent different cell types, and the size of outer nodes indicates the number of target genes involved in this cell type. (H) Violin plots showing the expression levels of core transcription factors *Xbp1* and *Atf3* in 1-, 2-, 5-, 12-, and 15-month-old mouse cochleae. (I) Ridge plots showing the gene set scores of *Xbp1* and *Atf3* target genes in 1-, 2-, 5-, 12-, and 15-month-old mouse cochleae. (J) Representative GO terms enriched for XBP1 target genes (top) and ATF3 target genes (bottom). Count indicates gene number.

Distinct from pairwise comparisons between two time points, multiple time nodes enable analysis of consecutive gene expression changes at high-temporal resolution, and identification of dynamic DEGs (DDEGs) (Zou et al., 2021; Fang et al., 2022). We, therefore, calculated gene expression trajectories along with the successive time points, and found that these can be clustered into six distinct dynamic patterns (Fig. 4A and Table S4). Each module contains genes with unique phase and magnitude along the trajectory. For example, in module 1, genes decrease in a nonlinear manner across time points, with a plateau between 12 and 15 months. Conversely, genes in module 6 steadily and persistently increase throughout life. Genes in module 2 or module 3 were gradually upregulated and decline at later time points, genes in module 4 displayed a cosine-like regulation while genes in module 5 rapidly decline in the first months and then increase rapidly in the aged animals (Fig. 4A).

We next sought to resolve steadily upregulated and downregulated DDEGs (module 6 or module 1) in different cell types throughout the entire temporal window, as such insight can inform mechanistic contributions to sustained and unidirectional effects on cochlear aging (Schaum et al., 2020). Based on the total number of DDEGs (module 6 or module 1), the top five cell types included IC, chondrocyte (CC), macrophage (M), Schwann cell (SC), and B cell (B) (Fig. 4B), suggesting their susceptibility to aging. We discovered that pronounced upregulated DDEGs were associated with four main functions: (i) protein folding (*Hsp90aa1*, *Calr*, and *Hspa5*), (ii) apoptotic signaling pathway (*Atf4*, *Ddit3*, and *Casp3*), (iii) positive regulation of adaptive immune response (*S100a8*, *S100a9*, and *Ifi35*), and (iv) response to oxidative stress (*Apoe*, *Sod2*, and *Gpx4*) (Figs. 4C, S8A and S8B), while pronounced downregulated DDEGs were associated with cell-substrate adhesion (*Otoa*, *Col3a1*, and *Actg1*), extracellular matrix organization (*Aebp1*, *Serpinh1*, and *Col2a1*), rhythmic process (*Dbp*, *Per3*, and *Nr1d1*), and cation transmembrane transport (*Kcnj13*, *Slc25a4*, *and Atp1a1*) (Figs. 4D, S8A and S8B).

To further delineate gene expression changes across all cell types during the aging process, we identified a total of 103 DDEGs that were consistently upregulated in at least five cell types, along with 88 genes consistently downregulated in at least four cell types (Fig. 4E), several of which have been experimentally validated by immunostaining (Figs. S9A–C). The top 10 frequently upregulated genes included *H2-K1*, *B2m*, *H2-D1*, *Isg15*, *Bst2*, *Hsp90aa1*, *Hsp90ab1*, *Bst2*, *Ifi27l2a*, *Mt2*, *S100a8*, and the top 10 shared downregulated genes were *Rbm3*, *Dct*, *Sparc*, *Dbp*, *Coch*, *Col1a2*, *Atp1b1*, *Ccnd2*, *Ptgds*, and *Car14* (Fig. 4E). Notably, most of the shared DDEGs showed similar function to that of total DDEGs (Fig. S9D and S9E). For example, shared upregulated DDEGs were correlated with protein folding (Fig. S9D and S9E). In addition, we performed an integrative comparative analysis of DDEGs with aging-associated genes from the Aging Atlas database (Aging Atlas, 2021). Interestingly, the top aging-high-risk genes encoded the chaperones HSP90AA1, HSPA1B, HSPA1A, and HSPA8 (Fig. 4F), whose consistent upregulation further points to an age-associated increased physiological requirement for conformational folding and assembly of other misfolded macromolecules (Saibil, 2013). Interestingly, persistently upregulated and downregulated DDEGs in HCs and SGNs also demonstrated age-related damages that may facilitate cochlear degeneration (Fig. S9F and S9G). For instance, *Nsmce3*, an upregulated DDEG in HCs, encodes a subunit of the SMC5/6 complex that is involved in DNA damage response (van der Crabben et al., 2016). Among the upregulated DDEGs in SGNs, *Sncb* encodes a member of a small family of proteins that inhibit phospholipase D2, which is abundant in neurofibrillary lesions of patients with Alzheimer’s disease and has been reported to trigger oxidative stress and inflammatory responses in the aged retina (Hadrian et al., 2019).

To explore how aging-associated DDEGs are regulated, we carried out a transcriptional regulatory network analysis across all the cochlear cell types (Fig. 4G). Among the top transcriptional regulators modulating upregulated DDEGs, a few were related to immune response including *Cebpb* (CCAAT enhancer binding protein beta) and *Stat3* (signal transducer and activator of transcription 3), *Irf7* (Interferon regulatory factor 7), whereas others were involved in the UPR, such as *Xbp1* (X-Box binding protein 1), *Atf4* (Activating transcription factor 4), and *Ddit3* (DNA damage inducible transcript 3), and apoptosis, including *Atf3* (activating transcription factor 3) (Fig. 4G). As for transcriptional modulators of downregulated DDEGs, *Dbp* (D-Box-binding PAR BZIP transcription factor), *Nr1d1* (nuclear receptor subfamily 1 group D member 1), and *Nr1d2* (nuclear receptor subfamily 1 group D member 2) are involved in circadian rhythm regulation (Fig. 4G). Among upregulated transcriptional regulators, we noticed that ER stress and apoptosis-associated transcription factors (TFs) *Xbp1* and *Atf3* as well as their target genes increased in an age-dependent manner (Fig. 4H–J), suggesting that these are core factors may contribute to the corresponding age-related phenotypes.

### Elevated UPR in aged intermediate cells

**Figure 5.**
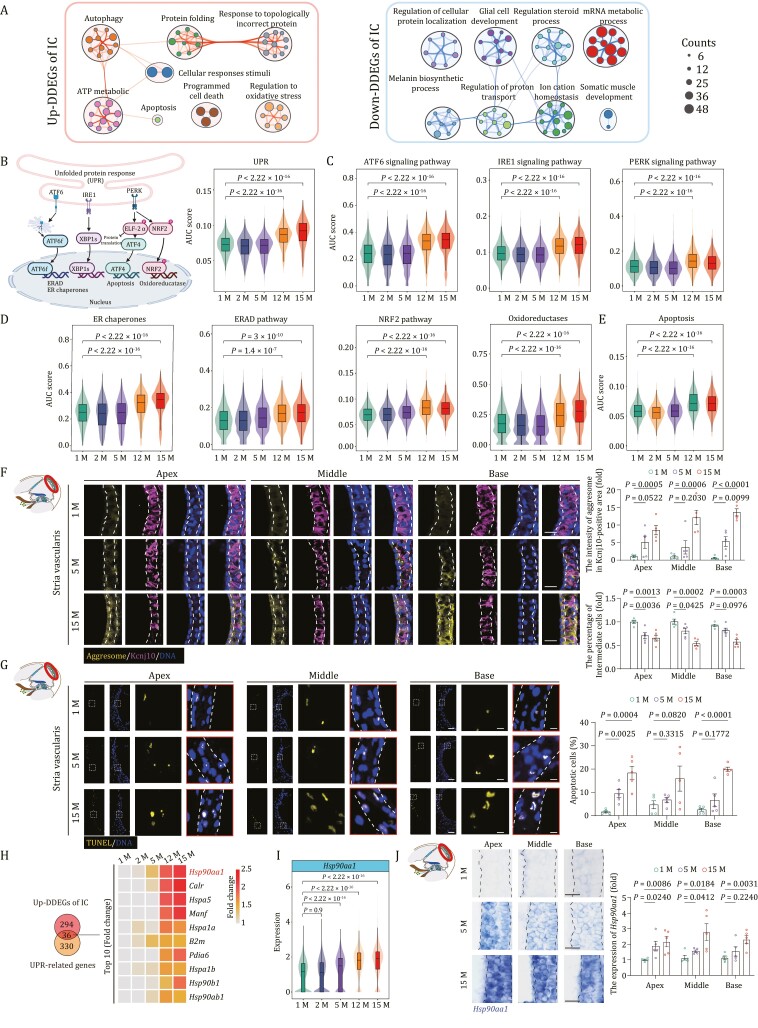
**Transcriptional profiles of intermediate cells during aging.** (A) Network graph visualizing representative GO terms and pathways of upregulated DDEGs (left) and downregulated DDEGs (right) in intermediate cells. The size of the node is proportional to the total number of hits that fall into that specific term. Two terms with similarity > 0.3 are connected by a line. (B) Left, a schematic showing three principal branches and corresponding core components in the UPR pathway. Right, Violin and box plot showing the gene set scores of UPR pathway in intermediate cells across different time points. Box shows the median and the quartile range (25%–75%) and the length of whiskers represents 1.5× the IQR. *P* values by Wilcoxon test are indicated. (C) Violin and box plots showing the gene set scores of ATF6, IRE1, and PERK signal pathways in intermediate cells from 1-, 2-, 5-, 12-, and 15-month-old mice. (D) Violin and box plots showing the gene set scores of ER chaperones, ERAD and NRF2 pathways, oxidoreductases in intermediate cells from 1-, 2-, 5-, 12-, and 15-month-old mice. (E) Violin and box plot showing the gene set scores of apoptosis in intermediate cells from 1-, 2-, 5-, 12-, and 15-month-old mice. (F) Co-staining of aggresome and Kcnj10 in the 1-, 5-, and 15-month-old cochlear samples. Scale bars, 20 μm. The relative intensity of aggresomes in Kcnj10-positive areas and the relative number of Kcnj10-positive cells are quantified as fold changes and presented as the mean ± SEMs (*n* = 5 mice). Two-tailed Student’s *t*-test *P* values were indicated. (G) TUNEL staining in the 1-, 5-, and 15-month-old cochlear samples. Scale bars, 20 and 5 μm (zoomed-in images). The percentage of apoptotic cells was presented as the mean ± SEMs (*n* = 5 mice). Two-tailed Student’s *t*-test *P-*values were indicated. (H) Left, Venn diagram showing an overlap between upregulated DDEGs in intermediate cells and UPR-related genes. Right, heatmap showing the expression levels of the top 10 (accumulated fold changes) overlapping genes. (I) Violin and box plot showing the expression levels of *Hsp90aa1* in intermediate cells from 1-, 2-, 5-, 12-, and 15-month-old mice. (J) RNA-ISH of *Hsp90aa1* in cochlear SV from 1-, 5-, and 15-month-old mice. Representative images are shown on the left; the relative intensity of *Hsp90aa1* is quantified as fold changes relative to that of apical turn in 1-month-old cochlea (*n* = 5 mice). Two-tailed Student’s *t*-test *P-*values were indicated. The values are shown as mean ± SEMs on the right. Scale bars, 20 μm. Month, M.

Based on our analysis, IC located in SV emerged as a cell type with dramatic perturbations in transcriptome. When we resolved the function of DDEGs in ICs, we found a negative correlation with cellular sodium ion homeostasis, melanin biosynthetic process, and gap junction assembly, suggesting compromised IC functions during aging (Fig. 5A) (Kim et al., 2013; Zhao, 2016). Consistent with previous findings, upregulated DDEGs in ICs had a positive relevance with UPR-associated pathways, including those in response to topologically incorrect protein, the HSP90 chaperone cycle, and apoptosis (Fig. 5A). When we analyzed the expression scores of UPR-associated genes, and the scores for the components in three major branching pathways of UPR, including ATF6, IRE1, and PERK signaling (Table S3), we found that all were consistently upregulated with age (Fig. 5B and 5C). Concurrently, we also examined gene scores for ER chaperones, ER-associated degradation (ERAD), nuclear factor erythroid 2-related factor 2 (NRF2) pathways and oxidoreductases (Fig. 5D and Table S3), which are the adaptive downstream cascades for the three major signaling pathways and can safeguard cells by either promoting protein (re)folding, degradation, or elicitation of antioxidant response (Xu et al., 2005; Wang et al., 2018; Gonzalez-Teuber et al., 2019; Li et al., 2020a). In this regard, the scores for all the downstream cascades of UPR were consistently upregulated with age in ICs (Fig. 5D and Table S3). Meanwhile, the gene set score for the apoptotic pathway, the principal cascade for PERK signaling was also upregulated with age (Fig. 5E and Table S3), suggesting that the adaptive UPR was not sufficient to counteract the unremitting ER stress and failed to reverse the age-related impairments in aged cochlea. In line with these findings, aggresome fluorescence intensity in ICs (Kcnj10^+^) and the proportion of TUNEL-positive cells markedly increased in SV tissues from 5- and 15-month-old mouse cochleae relative to those in 1-month-old mice (Fig. 5F and 5G).

As potentially critical regulators underlying the age-dependent defects, we discovered 36 shared genes between upregulated DDEGs and UPR-related genes, including a variety of chaperone-encoding genes *Hsp90aa1*, *Hspa5*, *Hspa1a*, *Hsp90b1*, and *Hsp90ab1* (Fig. 5H). Hence, chaperone-mediated protein refolding may represent one of the core adaptation mechanisms triggered by ER stress in aged ICs. *Hsp90aa1* (heat shock protein 90 alpha family class A member 1), ranked as the top one, was one of the most frequently occurring upregulated genes overlapped by DDEGs and annotated genes in Aging Atlas database as mentioned earlier (Figs. 4F, 5H, 5I, and S10A). In support of the gene expression data, RNA *in situ* hybridization (RNA–ISH) and Western blot assays also demonstrated the age-dependent elevation of HSP90AA1 levels (Figs. 5J and S10B). Altogether, these findings revealed that *Hsp90aa1*, an ER chaperone-coding gene, was the most commonly upregulated PDEGs and DDEGs in mouse cochlea and particularly in aged ICs, which suggests that targeting *Hsp90aa1* directly or indirectly could be a therapeutic strategy for mitigating cochlear aging-related dysfunction.

### Upregulation of chaperon HSP90AA1 alleviates the ER stress damages in SV cells

**Figure 6.**
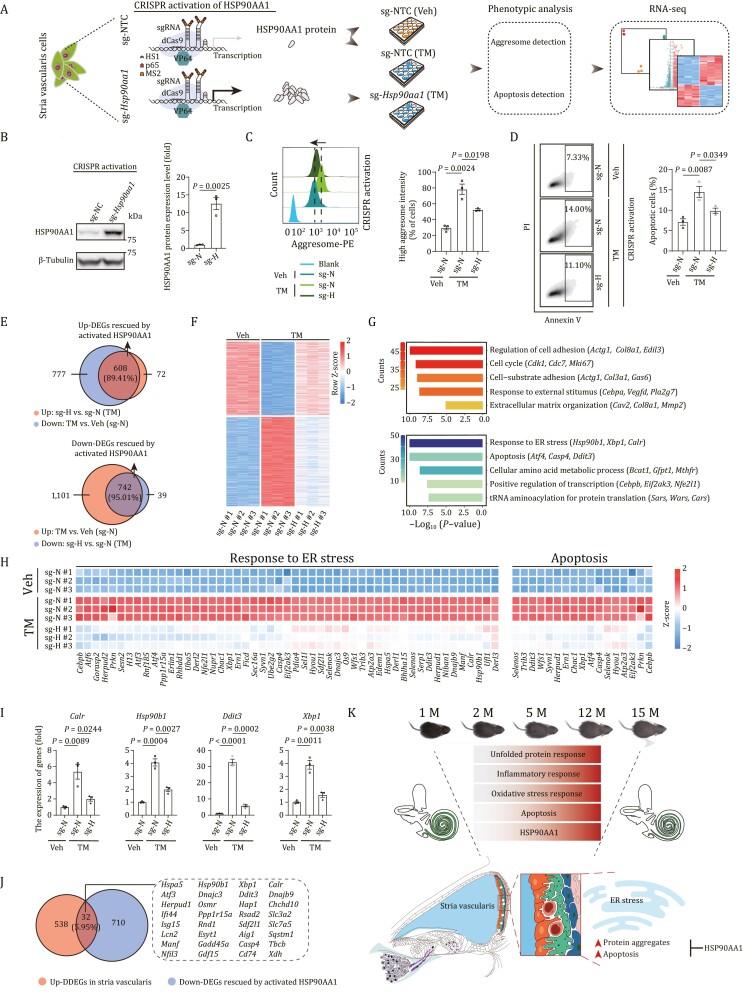
**Activation of HSP90AA1 prevents loss of proteostasis and alleviates apoptosis in stria vascularis cells.** (A) Schematic of CRISPR-dCas9 transcriptional activation system-based HSP90AA1 activation and phenotypic and mechanism analysis. NTC, non-targeting control. Veh, vehicle; TM, Tunicamycin. (B) Western blot and band intensity quantification of HSP90AA1 protein levels in stria vascularis cells (SV-K1) transduced with non-targeting or *Hsp90aa1*-targeting sgRNA. Data are presented as the mean ± SEMs, *n* = 3 biological repeats. Two-tailed Student’s *t*-test *P-*value was indicated. Representative data from one of the three independent experiments. sg-N, sg-NTC; sg-H, sg-*Hsp90aa1*. (C) Left, aggresome intensity analysis of stria vascularis cells transduced with non-targeting or *Hsp90aa1*-targeting sgRNA after treatment with vehicle or tunicamycin. Right, data are presented as mean ± SEMs, *n* = 3 biological repeats. Two-tailed Student’s *t*-test *P-*values were indicated. Representative data from one of the three independent experiments. (D) Left, apoptosis analysis of stria vascularis cells transduced with non-targeting or *Hsp90aa1*-targeting sgRNA after treatment with vehicle or TM. Right, the percentages of apoptotic cells are presented as mean ± SEMs, *n* = 3 biological repeats. Two-tailed Student’s *t*-test *P-*values were indicated. Representative data from one of the three independent experiments. (E) Venn diagram showed the number and percentage of upregulated (top) and downregulated (bottom) DEGs rescued by activated HSP90AA1 upon TM treatment. (F) Heatmap showing the expression levels of upregulated and downregulated DEGs rescued by activated HSP90AA1 upon TM treatment. (G) Representative GO terms of upregulated (top) and downregulated (bottom) DEGs rescued by activated HSP90AA1. Count indicates gene number. (H) Heatmaps showing the relative expression levels of genes related to response to ER stress and apoptosis in different groups. (I) The relative expression levels of indicated genes by RT-qPCR. Data are presented as mean ± SEMs, *n* = 3 biological repeats. Two-tailed Student’s *t*-test *P* values were indicated. A representative data from one of the three independent experiments. (J) The Venn diagram showing the genes shared by downregulated DEGs rescued by activated HSP90AA1 and upregulated DDEGs of stria vascularis. (K) Schematic illustration of the phenotypic changes and molecular mechanism of cochlear aging in mice.

To explore the potential of a chaperon-based mechanism, we asked whether genetic manipulation of *Hsp90aa1* could alter ER stress-induced phenotypes in the mouse cochlear SV cells (Figs. 6A and S10C). First, to mimic a physiological change that resembles mechanisms that might occur in cochlear aging *in vitro*, we treated SV cells with the canonical ER stressor tunicamycin (TM) (Zhang et al., 2014), and found that TM treatment resulted in an elevated stress response featured by increased levels of misfolded protein aggregates and apoptosis (Figs. 6C, 6D and S10D–F), which were exacerbated upon silencing of *Hsp90aa1* by CRISPR-mediated knockdown (Fig. S10G–I). Conversely, when we induced endogenous HSP90AA1 expression in SV cells using a CRISPR-dCas9 transcriptional activation system (Fig. 6B) (Joung et al., 2017; Hu et al., 2020), we noticed that the elevated levels of protein aggregates and apoptosis induced by TM challenge were alleviated (Fig. 6C and 6D). Furthermore, RNA-seq analysis also indicated that HSP90AA1 activation reversed the TM stress-induced transcriptional profile, and generated a gene expression profile more closely resembling that of the TM-untreated state (Figs. 6E, 6F, S10J and S10K; Table S5). For instance, the escalated expression levels of genes such as the core UPR TFs-coding genes *Atf4*, *Xbp1*, and *Atf6*, chaperons-coding genes *Hyou1*, *Hsp90b1*, and *Calr*, associated with ER stress, *Ddit3* and *Atf4*, associated with apoptosis, were repressed in HSP90AA1-activated SV cells (Fig. 6G and 6H). To confirm the RNA-seq results, we validated the restored expression of a few of the key affected genes involved in response to ER stress (*Calr*, *Hsp90b1*, *Ddit3*, and *Xbp1*) by RT-qPCR (Fig. 6I). Finally, we asked whether the transcriptional changes we had detected in SV cells *in vitro* were similar to those in mouse SV cells during cochlear aging. Indeed, we identified 32 genes that were shared between downregulated genes upon HSP90AA1-activation and upregulated DDEGs in SV cells, including a panel of genes encoding UPR-related chaperons or TFs (*Hspa5*, *Hsp90b1*, *Xbp1*, *Calr*, *Atf3*, *Dnajc3*, *Ddit3*, etc.) (Fig. 6J). In conclusion, our findings demonstrate that HSP90AA1 activation counteracts deleterious effects of ER stress in SV cells by dampening the protein aggregates and accompanying cellular apoptosis.

## Discussion

Age-dependent and progressive functional deterioration in cochlea is a known component of ARHL, however, as for most degenerative processes, its underlying and potential molecular mechanisms have remained largely elusive. In the present study, we have constructed a single-cell transcriptome landscape of mouse cochlear aging. Our analysis spans five cochlear regions across five time points for which we identified 27 cell types based on their unique transcriptional profiles. By using pairwise and dynamic differential expression analysis, we found that cochlear aging is characterized by increased ER and oxidative stress, and elevated inflammation and apoptosis in a variety of cochlear cells. Among them, we discovered that ICs in the SV are a cell population with the highest CV along with the most PDEGs and DDEGs, and with gradual and persistent upregulation of a compensatory ER chaperon-coding gene *Hsp90aa1*. In functional experiments, activation of HSP90AA1 can alleviate ER stress-induced damages. In summary, our work provides a rich resource for mining cellular and molecular mechanisms of cochlear aging at high temporal and single-cell resolution, and for advancing development of diagnostic and therapeutic interventional strategies against ARHL (Fig. 6K).

Age-related functional deterioration in cochlea is associated with a range of hearing disorders, placing the cochlea, a multicompartmental and complex structure, at the forefront of both fundamental biological research and translational neurobiology. Although several pioneering studies described the cell composition of the cochlear sensory epithelium, sensory neurons, and SV at single-cell resolution (Burns et al., 2015; Waldhaus et al., 2015; Ellwanger et al., 2018; Petitpre et al., 2018; Shrestha et al., 2018; Sun et al., 2018; Korrapati et al., 2019; Zhu et al., 2019; Kolla et al., 2020; Janesick et al., 2021; Kubota et al., 2021; Milon et al., 2021), a tissue-level transcriptional profiling analysis that reveals the full cellular complexity and cell type-specific molecular properties across the entire cochlea was lacking. By utilizing an optimized single-cell disassociation procedure (see Methods), we have established a single-cell transcriptome atlas for mouse cochlea that captures a broad spectrum of cell types from five major regions, including the organ of Corti, the modiolus, the Reissner’s membrane, the SV, and the spiral ligament across five time points. This atlas broadens our understanding of cell identities and cell type-specific signatures in mouse cochlea, and lays a foundation for investigating the cellular and molecular changes in physiological and pathological conditions. Due to the known technological difficulties associated with isolating mature neurons with intact dendrites and axons, we only captured a small number of SGNs (Petitpre et al., 2018; Shrestha et al., 2018; Sun et al., 2018). It is possible that single-nucleus RNA sequencing and spatial transcriptomic analysis are technologies better suited to capture hard-to-detect or even undetectable cell types (He et al., 2020; Dar et al., 2021; Zhang et al., 2021), and allow for measuring their transcriptional profiles. Another notable caveat for studying cochlear aging is the scarcity in the number of HCs, which serves as technical restriction impeding a comprehensive analysis in this study as well as in others (Chen and Corey, 2002; Gong et al., 2006; Barsh et al., 2018). Therefore, genetic labeling with GFP followed by fluorescence-activated cell sorting (FACS) or manually picking to enrich HCs or SGNs before subjecting them to in-depth scRNA-seq might be required (Waldhaus et al., 2015; Barsh et al., 2018; Petitpre et al., 2018; Ranum et al., 2019; Milon et al., 2021; Liu et al., 2022).

In comparison with other mouse strains or animal species, C57BL/6J mice recapitulate relatively more aspects of human ARHL including the dynamics of hearing loss and the pattern of cellular degeneration (Li and Borg, 1991; Le Calvez et al., 1998; Zheng et al., 1999; Popelar et al., 2003; Keithley et al., 2004; Fetoni et al., 2011; Seo et al., 2021; Xue et al., 2021). By using this model, we determined the dynamics of cochlear aging at a high-temporal resolution by pairwise and dynamic analyses of transcriptional profiles, and found that age-related gene expression alterations in the mouse first appeared around 2 months of age, whereas dramatic changes were already present at the fifth month, and then persisted until the 15th month, which was in line with the phenotypic characteristics of the C57BL/6J mouse model (Hequembourg and Liberman, 2001; Kane et al., 2012; Mellado Lagarde et al., 2014; Zhang et al., 2015). Of note, we identified ICs in the SV with the highest CV at each time point, alongside the most age-dependent DDEGs throughout life. This finding was consistent with the notion that stria presbycusis exhibits slow and progressive hearing loss probably due to SV atrophy and subsequential dysfunction of endolymphatic metabolism, exacerbating accumulation of age-related damages (Suzuki et al., 2006; Yang et al., 2013). Our data not only unveiled the transcriptomic changes underlying the onset time and progression of ARHL at high-temporal resolution and in a cell type-specific manner, but also suggested a crucial role of SV whose dysfunction may underlie presbycusis. However, it should be noted that C57BL/6J mice may have limitations as a model for cochlear aging research, such as carrying *Cdh23* mutation. In the future, better models more resembling human ARHL (e.g., non-human primates) are in urgent need (Zou et al., 2022).

In the present study, we demonstrated that escalated inflammation and oxidative stress as well as apoptosis are key features across the timeline of cochlear aging, which was supported by prior studies (Staecker et al., 2001; Su et al., 2020). Notably, we identified UPR as a prominent feature of cochlear aging, evidenced by a series of persistently elevated adaptive and apoptotic UPRs. Adaptive UPRs included increased expression of a large number of ER chaperons such as heat shock family proteins, as well as core components of the ERAD pathway in the aged ICs, which help to enhance the protein folding capacity and remove misfolded proteins (Xu et al., 2005; Mori, 2009; Urra et al., 2016; Li et al., 2020a). In addition, we found a persistently elevated PERK pathway in the aged ICs, which can repress mRNAs translation and reduce the influx of new proteins into the ER. These findings together suggest that several compensatory mechanisms that to some extent defy aging pressure may co-exist in the cochlea. Although cells initially aim to compensate for damage through those adaptive pathways, the extent of apoptosis we observed in the aged cochlea implies that excessive and prolonged ER stress triggered cell death to dispense of dysfunctional cells (Xu et al., 2005; Zhang and Kaufman, 2006; Mori, 2009; Walter and Ron, 2011; Read and Schroder, 2021). Ultimately, we surmise that an aging-associated mechanism that leads to elevation of physiological UPR levels can only compensate but not prevent cochlear aging. Although a previous study found a decrease in the expression of GRP78 as a crucial molecular chaperon in aged cochlea, they also observed that ubiquitinated proteins and apoptotic cells accumulated in the aged cochlea (Wang et al., 2015). To further untangle the regulatory mechanism of UPR underlying the cochlear aging, in-depth mechanistic investigations are needed in the future.

Interestingly, we identified ER chaperon-coding gene *Hsp90aa1* as one of the top upregulated genes during cochlear aging. Although HSP90AA1 is a known stress-inducible protein that aids protein folding to maintain cell homeostasis, to our knowledge, it has not previously been linked to cochlear IC aging. Under the pressure of noise, HSP90AA1 has shown a certain protective effect on the cochlea (Jongkamonwiwat et al., 2020), suggesting a plausible protective role in other auditory pathological conditions. In addition, upregulation of *HSP90AA1* was also observed in aged human skin melanocytes and primate immune cells (Aging Atlas, 2021), strengthening the potential connection between HSP90AA1 and aging. Indeed, a study in rats showed that metformin, a well-known drug shown to delay aging and extend health span in animals, attenuated d-galactose-induced aging-associated hearing loss by reducing the expression of ER stress-associated proteins including HSP90, HSP60, CHOP, and GRP78 (Cai et al., 2020). Intriguingly, HSP90AA1 was recently reported to function as a secretory protein involved in many biological processes (Kim et al., 2021). Whether any diagnostic or targeted intervention strategies for presbycusis can be developed based on HSP90AA1 requires further investigation.

In summary, by constructing a single-cell resolution transcriptomic atlas of mouse cochlear aging, we provided a resource for advancing in-depth understanding of mechanisms underlying physiological aging of a complex auditory organ. We identified that loss of proteostasis is a hallmark of cochlear aging, thus selective targeting of the UPR pathway or its core regulators may pave the way toward development of therapeutic approaches for delaying the onset of physiological cochlear aging and presbycusis.

## Methods

### Animals

C57BL/6J mice used in this study were purchased from SiPeiFu (Beijing) Biotechnology Co. Ltd. and kept in a quiet environment with enough water and food under a standard 12:12-h light-dark (LD) cycles with 22°C and 55% humidity.

### Cells

SV cells (SV–k1) were purchased from Bluefbio in China. Cell culture was carried out as reported previously (Gratton et al., 2002). In brief, the cells were cultured in High glucose medium (Hyclone, sh30022.01) supplemented with 10% fetal bovine serum (FBS) (GIBCO, 10270-106), 100 U/mL penicillin and 10 mg/mL streptomycin (Thermo Fisher Scientific) in permissive growth condition. 80% confluent cultures were detached with 0.25% Tryple (Gibco, 12605010) and then split at a ratio of 1꞉5. All the cell cultures were tested negative for mycoplasma contamination. Cells were cultured under permissive conditions to 80% confluence, then transferred to nonpermissive conditions for 24 h, followed by treatment with 1 μg/mL TM for 2 h and replaced with fresh culture medium. 16 h later, apoptosis levels and fluorescence intensity of aggresome were analyzed.

### ABR

The ABR threshold is determined as described previously (Fu et al., 2018). Briefly, mice were deeply anesthetized by intraperitoneal injection of pentobarbital sodium (50 mg/kg body weight). The temperature of the mice was maintained at 37°C using a heating pad in an acoustic shielding chamber. Output stimuli were calibrated at the beginning of experiment with a one-quarter inch microphone (PCB Piezotronics model PCB-378C01; or Bruel and Kjær, 4939A011 and 2690A0S1) positioned where an experimental animal’s ear would be. Three silver wire electrodes were inserted into the skin to record the responses: the active electrode was inserted into the top of the skull between the ears of the mice, the reference electrode was inserted under the right ear of the mice, and the ground electrode was inserted into the dorsal midline. Tone burst stimuli (8, 12, 16, 24, and 32 kHz) were presented at a rate of 21.1 per second, and responses were recorded using a Tucker-Davis Technologies System (TDT, USA) workstation running BioSigRZ software. The intensity of the input stimulus was initially set at 90 decibels sound pressure level (dB SPL) and then decreased every 5 dB until the threshold level was reached. The threshold was defined as the lowest sound intensity at which the reproducible waves were visually identifiable. Blinding procedures were applied to hearing assessment across different ages in mice.

### DPOAEs

DPOAE responses of 2f1-f2 were measured using the Real-time Signal Processing System II from Tucker-Davis Technologies as described previously (Fu et al., 2018). Output stimuli were calibrated as in ABR measurement. Two level (L1 = 80 dB SPL, L2 = 75 dB SPL) primary signals (f1 and f2), with f2/f1 = 1.22. DPOAE response thresholds were recorded across a range of frequencies (8, 12, 16, 24, and 32 kHz). The primary tones produced by two separate speakers (EC1 closed-field speakers, TDT), DPOAE recordings were made with a low-noise microphone ER 10B (Etymotic Research, Elk Grove Village, IL). A peak at 2f1-f2 in the spectrum was accepted as a DPOAE if it was 3 dB above the noise floor. Blinding procedures were applied to hearing assessment across different ages in mice.

### Endocochlear potential determination

Endocochlear potential determination was performed as described previously (Mei et al., 2017). Briefly, after anesthesia, the mouse cochlea was exposed by a ventral approach. Then, the tympanic bulla was opened, tissue and muscle covering the bulla were carefully removed, and a small opening was made with a small pick. A glass capillary microelectrode filled with 150 mmol/L KCl was installed on a Leica micromanipulator. The ground electrode was inserted into the dorsal neck muscle and the microelectrode was inserted into the middle stage through the lateral wall of the cochlear duct. The response from the microelectrode was amplified using an Axopatch 200 B amplifier in current clamp mode and captured using pClamp 10 software.

### Paraffin-embedded and frozen sections

After sacrifice by cervical vertebrae dislocation, the temporal bones were dissociated from mouse inner ear, a hole on the apical cochleae was made and tissue was fixed in 4% paraformaldehyde (Dingguo, AR-0211) in PBS for about 24 h at 4°C, followed by three washes with PBS and decalcification with 10% EDTA for 72 h at 4°C to completely soften the cochlea. For paraffin-embedded sections, the cochleae were dehydrated by gradient alcohol and then embedded in paraffin, and cut into 5 μm sections. For the frozen sections, the completely decalcified cochleae were dehydrated with 15% sucrose solution and 30% sucrose solution and were embedded in optimal cutting temperature (OCT) compound (Sakura Finetek, 4583), snap-frozen in liquid nitrogen and then stored at −80°C. Frozen samples were cut into 8-μm sections using a CM 1850 cryostat (Leica Microsystems Nussloch).

### H&E staining

H&E staining was performed as previously described (Kharkovets et al., 2006; Frisina et al., 2016). Slides were placed in a series of clearing xylene solutions for 30 min and rehydrated in a graded series of ethanol (100%, 100%, 95%, 80%, 70%, and 50%) and briefly washed in distilled water. The slides were then incubated with hematoxylin (Service bio, China) for 5 min, and washed with running tap water to remove excess hematoxylin followed by differentiation in 1% acid alcohol for 10 s and washed with running tap water for 1 min. This step was followed by an incubation in the eosin counterstain, subsequent dehydration in a graded series of ethanol (80%, 95%, 95%, 95%, 100%, and 100%), and immersion in xylene. At last, the slides were cover-slipped with resinous mounting medium.

### Tissue immunostaining

Frozen sections were washed in PBS for 5 min and then fixed by 4% paraformaldehyde (Dingguo, AR-0211) in PBS for 25 min at room temperature (RT). Paraffin-embedded sections were deparaffinized in xylene and rehydrated through gradient alcohol (100%, 100%, 90%, 80%, 70%, and 50%). After rinsing in distilled water, antigen retrieval was performed by microwaving the slides in 10 mmol/L sodium citrate buffer (pH 6.0) three times for 5 min each. Upon cooling down to RT, the sections were rinsed in PBS for three times. Then frozen sections or paraffin-embedded sections were permeabilized and blocked for 1 h at RT in PBS with 0.4% Triton™ X-100 (Sigma-Aldrich, T9284) and 10% donkey serum in PBS, respectively. Sections were then incubated with the appropriate primary antibodies (Rabbit anti-Neutrophil Elastase, Abcam, ab21595; Rabbit anti-4-Hydroxynonenal, Abcam, ab46545; Rabbit anti-β-Tubulin III (Tuj1), Sigma, T2200 Rabbit anti-myosin 7a, Proteus Bioscience, 25-6790; Rabbit anti-CALR, Abcam, ab92516; Rabbit anti-MRP8, Abcam, ab180735; Rabbit anti-S100A9, Abcam, ab92507) in 10% donkey serum overnight at 4°C, followed by incubating with secondary antibodies and Hoechst 33342 (Thermo Fisher Scientific, H3570) for 1 h at RT. After additional several washes, the sections were mounted with VECTASHIELD Antifade Mounting Medium (Vector Laboratories, H-1000), and images were obtained with a confocal laser-scanning microscope (Zeiss 900 confocal system).

### Aggresome staining

Aggresome staining was performed following the manufacturer’s protocol (Enzo, ENZ-51035-K100). Frozen sections were fixed by 4% paraformaldehyde (Dingguo, AR-0211) in PBS for 15 min followed by permeabilization with 0.3% Triton™ X-100 (Sigma-Aldrich, T9284) for 7 min, incubated with the Aggresome dye (1:5,000 dilution in PBS) for 3 min and then destained in 1% acetic acid for 30 min. The sections were then blocked with 10% donkey serum in PBS for 1 h after washing thoroughly with PBS and followed by incubation with rabbit anti-Kcnj10 antibody (Alomone, APC-035, 1:200) overnight at 4°C. The sections were then counterstained with Hoechst 33342 (Thermo Fisher Scientific, H3570) and fluorescence-labeled secondary antibody (Thermo Fisher Scientific, A10042) for 1 h at RT after washing with PBS for 3 times (10 min each). Image was obtained by Confocal laser-scanning system (Zeiss 900 confocal system). The aggresome intensity was quantified by Image J software and normalized to that of 1 month old mice.

### TUNEL staining

TUNEL (terminal deoxynucleotidyl transferase dUTP nick end labeling) staining was conducted on paraffin sections utilizing the Kit (Beyotime, C1088) following the manufacturer’s protocol. Then, the slides were counterstained with Hoechst 33342 (Thermo Fisher Scientific, H3570) for visualization of nucleus. Finally, the slides were mounted with VECTASHIELD Antifade Mounting Medium (Vector Laboratories, H-1000) and the percentages of TUNEL-positive cells were quantified by Image J software. The number of TUNEL-positive cells was normalized to that of 1 month old mice.

### RNA-ISH

RNA-ISH was carried out as reported previously (Ma et al., 2021). In brief, the mouse cochleae were quickly harvested by dissection after the mice were sacrificed and then were fixed for about 48 h with 5% paraformaldehyde at 4°C. The fixed cochleae were dehydrated by 15%, 30% sucrose solution, and embedded in OCT compound for cryoprotection. The thickness of frozen sections for RNA–ISH is 10 μm and the primers used to clone the *Hsp90aa1* fragment from mouse cDNA and subsequent labeling of the RNA probe were as follows: Forward: gaggaaacccagacccaaga, Reverse: gatcccccagctgaggactc. DIG RNA Labeling Mix (Roche Diagnostics) were used to transcribe DIG-labeled RNA probes by T7 and T3 RNA polymerases. Then, RNA–ISH was performed following a previously described method (Schaeren-Wiemers and Gerfin-Moser, 1993). In brief, frozen sections were washed in PBS for 10 min and then treated by prehybridization buffer overnight at RT and then incubated with hybridization mixture containing DIG-labeled RNA probes targeting *Hsp90aa1* overnight at 65°C. The slides were blocked for 1 h and incubated for 1 h with anti-DIG antibody at RT. Finally, the color reaction was performed and sections were sealed with glycerin. The image was captured by optical microscope and analyzed by ImageJ software.

### Flow cytometric analysis

#### Apoptosis analysis

The detection of cellular apoptosis was carried out in accordance with the manufacturer’s instructions (Vazyme Biotechnology, A211-02). Briefly, the freshly collected cells were stained with Annexin V-EGFP Apoptosis Detection Kit and then analyzed by BD LSRFortesa flow cytometer.

#### Aggresome intensity analysis

The analysis of cellular aggresome intensity was performed following the manufacturer’s instructions. The freshly collected cells were stained by PROTEOSTAT® Aggresome detection kit (ENZO, ENZ-51035-K100) and then analyzed by BD LSRFortesa flow cytometer (Wang et al., 2018).

### Knockdown and induction of endogenous expression of HSP90AA1 by using CRISPR/CAS9 system

The CRISPR/Cas9-mediated gene knockdown and activation were performed as previously described (Sanjana et al., 2014; Hu et al., 2020; Wang et al., 2021b). For knockdown of *Hsp90aa1*, guide RNA targeting *Hsp90aa1* and two non-targeting controls (NTCs) was cloned into LentiCRISPRv2 vector (Addgene #52961). For activation of endogenous expression of HSP90AA1, guide RNA targeting the transcriptional start site (TSS) locus of *Hsp90aa1* and two NTCs were constructed into lentiSAM v2 vector (Addgene #75112). To produce lentivirus particles, HEK293T cells were co-transfected with transfer plasmid lentiviral sgRNA plasmids or lentiMPH v2 (Addgene #89308), along with packaging plasmids psPAX2 (Addgene #12260) and pMD2.G (Addgene #12259) by Lipofectamine 3000 transfection reagent (Invitrogen, L3000015). The supernatants containing viral particles were collected 48 and 72 h later and were ultra-centrifuged at 19,400 ×*g* for 2.5 h to obtain virus particles. To knockdown the expression of *Hsp90aa1*, SV cells were transduced with lentiviral vector LentiCRISPRv2 for 48 h in the presence of 10 μg/mL polybrene following selection with puromycin for 5 days. To transcriptionally activate the endogenous expression of HSP90AA1, SV cells were co-transduced with the produced lentiviral vectors LentiSAM v2 and LentiMPH v2 for 48 h in the presence of 10 μg/mL polybrene before selection with blasticidin and hygromycin for 5 days. To verify the successful knockdown or activation of HSP90AA1, the selected cells were collected for Western blot to test the protein levels of HSP90AA1.

### Western blot

Cells were lysed with SDS lysis buffer [containing 4% SDS and 100 mmol/L Tris-HCl (pH = 6.8)] and heated at 105°C for 10 min. Mouse cochleae were grinded and then lysed with 200 μL SDS buffer for 24 h at 4°C followed by heating at 105°C for 10 min and then centrifuged at 21,100 ×*g* for 30 min to extract the supernatant for protein concentration quantification. Then the protein concentration of each sample was measured by BCA kit following manufacturer’s instruction. Each sample was electrophoresed by SDS–PAGE and then electrotransferred to PVDF membrane (Millipore) which was then blocked with 5% skimmed milk (powder from BBI Life Sciences) and incubated with the primary antibodies (Mouse anti-Hsp90 alpha, Abcam, ab79849; Mouse anti-Kcnj13, Santa, sc-398810; Mouse anti-β-Tubulin, Immunoway, YM3030) overnight at 4°C, and then incubated with the secondary antibodies conjugated with horseradish peroxidase (HRP). At last, the visualization and data processing were performed by a ChemiDoc XRS system (Bio-Rad).

### RNA isolation and analysis

RNA was extracted according to the manufacturer’s instructions after cells were lysed by TRIzol™ Reagent (Thermo Fisher Scientific, 15596018) and the concentration of RNA was measured after dissolving in about 20 μL DEPC water (Sangon Biotech). 2 μg RNA was subjected to reverse transcription according to the instructions of the GoScript™ reverse transcription system (Promega) to obtain cDNA. RT-qPCR was then performed with the qPCR Mix (TOYOBO) on a CFX384 RT-PCR system (Bio-Rad). The relative mRNA expression level of each gene was normalized to *Gapdh* expression, calculated using the ∆∆Cq method. The primers used for RT–qPCR are as follows: *Xbp1*-Forward, GACAGAGAGTCAAACTAACGTGG; *Xbp1*-Reverse, GTCCAGCAGGCAAGAAGGT; *Calr*-Forward, TGGCTGCTCCCAATAATGTCT; *Calr*-Reverse, GAGGGTAGTGACC AAAAGATGG; *Hsp90b1*-Forward, TCGTCAGAGCTGATGATGAAGT; *Hsp90b1*-Reverse, GCGTTTAACCCATCCAACTGAAT; *Ddit3*- Forward, CTGGAAGCCTGGTATGAGGAT; *Ddit3*-Reverse, CAGGGT CAAGAGTAGTGAAGGT; *Gapdh*-Forward, TGGATTTGGACGCAT TGGTC; *Gapdh*-Reverse, TTTGCACTGGTACGTGTTGAT.

For RNA-seq, 1.5 μg total RNA was provided to the Novogene Bioinformatics Technology Co. Ltd. for subsequent quality control, library construction and high-throughput sequencing. Briefly, Next Ultra RNA Library Prep Kit for Illumina (NEB) was used for sequencing library construction and the resultant libraries were sequenced on an Illumina paired-end sequencing platform by 150-bp read length.

### Single cell isolation

The samples for scRNA-seq included 1-, 2-, 5-, 12-, and 15-month-old mice and each age group contains 5 males and 5 females. The cochleae of mice of the same age and sex were pooled together for scRNA-seq. Briefly, the cochleae were taken out and placed in chilled 500 μL Leibovitz’s L-15 medium (Gbico, 11415064) quickly after mice were sacrificed by cervical dislocation. Afterward, temporal bones of mice were isolated, and the overlying bone was extracted, leaving the lateral wall, the modiolus with the spiral ganglion, the spiral limbus, the inner sulcus, the organ of Corti and the outer sulcus remaining. Microdissected tissues from each mouse were pooled and transferred into 500 μL digestion solution I containing Leibovitz’s L-15 medium (Gbico, 11415064), 200 unit/mL collagenase IV (Sigma, C1889) and 10 K unit/mL deoxyribonuclease I (DNase I, Sigma, DN25) and incubated at 37°C for 30 min, pipetted once every 10 min. Thirty minutes later, digestion solution I was changed to digestion solution II containing 1× EBSS (Gibco, 14155-063), 20 unit/mL papain (Sigma, P4762), 1 mmol/L l-Cysteine (Sigma, C1276), 0.5 mmol/L EDTA, 15 mmol/L HEPES (Aladdin, H109407), and 10 K units/mL DNase I (Sigma, DN25) at 37°C for 30 min, pipetted 10 times every 10 min. 50 μL 10% fetal bovine serum (FBS, Gibco, 10270-106) was added to terminate the digestion followed by pipetting 100 times in order to obtain single cell suspension. After a centrifugation with 1500 rpm at 4°C for 5 min, the cell pellet was resuspended and filtered with a 40-μm strainer (BD Falcon) and washed with 1 mL of L15 medium. Filtered cell suspension was collected directly into a 1.5 mL EP tube and Hoechst (Thermo Fisher Scientific, H3570), Propidium Iodide were added, followed by FACS (BD Influx) to remove the debris and dead cells. The obtained single cells were centrifuged with 2000 rpm at 4°C for 5 min and resuspended in 0.04% bovine serum albumin (BSA, Gibco) in PBS for scRNA-seq.

### Droplet-based scRNA-seq using the 10× Genomics chromium platform

Single cells were captured by droplet-based microfluidic technology and the construction of single cell transcriptional libraries was performed by the Chromium 10× Single-Cell Instrument (10× Genomics) and 10× Genomics Chromium Single Cell 3ʹ GEM Library and Gel Bead Kit v3. In brief, cells were loaded in each channel with a target output of 5000 cells per sample with appropriate cell concentration measured by Moxi GO II (Orflo Technologies). All the reactions were performed in the Bio-Rad C1000 Touch Thermal cycler with 96 Deep-Well Reaction Module in which 12 cycles were used for cDNA amplification and sample identification. Amplified cDNAs and final libraries were then evaluated on a Fragment Analyzer (AATI) using a High Sensitivity NGS Analysis Kit (Advanced Analytical). The average fragment length of the 10× cDNA libraries was assessed with the AATI, and quantified by qPCR using the Kapa Quantification kit. All the libraries were diluted and pooled together for each run of NovaSeq sequencing. All the libraries were sequenced on the NovaSeq 6000 Sequencing System (Illumina).

### Processing raw data from scRNA-seq of 10× Genomics

Cell Ranger single-cell software suite (version 3.1.0) (10× Genomics) with default parameters was used to align and quantify the dataset. The quantification of the sample-specific FASTQ file was evaluated by the *cellranger count* function, which was aligned to the mouse reference genome (mm10) and generate the gene expression matrix. The filtered gene expression matrix was used for downstream analysis.

### scRNA-seq data analysis and cell-type identification

Downstream analysis for scRNA-seq data was implemented by the single-cell toolkit Seurat (version 3.2.0) (Stuart et al., 2019) in R (version 3.6.3). In order to get high-quality cells, only cells with more than 500 genes and less than 5,000 genes detected, and less than 20% mitochondrial genes were used for subsequent analysis. In addition, in order to remove the “doublets” from the scRNA-seq data, we also used the R package DoubletFinder (version 2.0.3) (McGinnis et al., 2019) to identify and remove double cells in each sample. In order to generate enough artificial doublets, we set the pN value to 0.25. We combined the number of cells in this study and the doublets ratio of 10× Genomics single cell platform “results in recovery of ~1000 cells, and a multiple rate of ~0.8%”, and set the doublet formation rate as “doubletate = cellnum × 8 × 10^−6^”. The “find.pK” function was used to calculate the optimal pK value. After quality control, 45,972 cells remained and were used for downstream bioinformatic analyses. In order to better eliminate the false positive of biological heterogeneity caused by technical factors such as sequencing depth in scRNA-seq data, we normalized and scaled the data based on R package SCTransform (version 0.3.2) (Hafemeister and Satija, 2019) to reveal clearer biological differences. The “*PrepSCTIntegration*” and “*FindIntegrationAnchors*” functions were used to select integration anchors and perform downstream integration. Then these anchors were used to integrate the data set of all the samples together with “*IntegrateData*” function. The integrated data set was then used for downstream dimensionality reduction and clustering analyses. Total cell clustering was performed by “*FindClusters*” function at a resolution of 2.0 and the first 30 principal components (PCs) were used to define cell identity. Dimensionality reduction was performed with “*RunUMAP”* function. Marker genes for each cluster were determined with the Wilcoxon rank-sum test by “*FindAllMarkers”* function. Only those with “avg_logFC” > 0.5 and “p_val_adj” < 0.05 were considered as marker genes. Cell types were identified based on the expression of classic marker genes. Marker genes for each cell type are shown in Table S1.

### Determination of the purity of cell type

The algorithm called Ratio of Global Unshifted Entropy (ROGUE) provided by Zhang et al. (Liu et al., 2020) was occupied to accurately assess the purity of the identified cochlear cell types.

### Age-relevant CV analysis

Analysis of age-relevant CV was used to observe the aging effects on different cell types (Salzer et al., 2018; Wang et al., 2020b). The “*FindVariableGenes*’’ function of Seurat was used to identify highly variable genes (HVGs). The top 10% variable genes (2,095 out of 20,953 genes) were selected for downstream analysis. Next, the absolute value of the cell-paired-distance dc,x was calculated between each HVGs expression in all cells of the young individuals and the old individuals in each cell type c:


$$\begin{array}{l} \mu_{c,x}=\;\left|X_{c,j}-X_{c,j}\right|;i\in \;\{1,\;2,\;\&,\;y\},\;j\in \;\{1,2,\ldots ,0\} \end{array}$$

Finally, the arithmetic mean of dc,x (mc,x), and the standard deviation of dc,x as (sc,x) were calculated. Accordingly, the aging-related transcriptional variation of each cell type is defined by the following formula:


$$\begin{array}{l} CV_{c,x}=\;\frac{\sigma_{c,x}}{\mu_{c,x}}\times 100 \end{array}$$

### Identification of aging-associated PDEGs

We used the function of ‘‘*FindMarkers*’’ in Seurat to identify aging-associated PDEGs between 1-month-old and each consecutive time point. The log (fold change) (LogFC) and adjusted *P-*value of each PDEG were calculated by the non-parametric two-sided Wilcoxon rank-sum test and only those with |“avg_logFC”| > 0.25 and “p_val_adj” < 0.05 were considered to be aging-associated PDEGs. We also ran similar analyses using 2-month-old mice as reference and got PDEGs between neighboring ages. The PDEGs are listed in Table S2.

### Identification of age-dependent DDEGs

To identify age-dependent DDEGs, we borrowed the method of the “*plot_pseudotime_heatmap*” function in R package Monocle2 (Qiu et al., 2017) and customized a function. First, we used the function of “*FindMarkers*” in Seurat to identify DEGs of any age (1, 2, 5, 12, and 15 months of age) in each cell type. Only those genes with |“avg_logFC”| > 0.25 and “p_val_adj” < 0.05 are considered age-related DEGs. Second, for each cell type, we used the above genes to construct an expression matrix (row genes, column as cells), and then sort all cells by age scale (i.e., each column in the expression matrix was ranked according to the age rank corresponding to each cell). Next, we used the “*genSmoothCurves*” function to fit smooth spline curves for the gene expression matrix dynamics along age time in a gene-wise manner and return the corresponding response matrix. Finally, the “ward. D2” method was used to cluster the matrix by row hierarchical clustering through “hclust” function, and the clustering results were assigned to 8 groups. The R package pheatmap (version 1.0.12) was used to visualize the age-dependent genes expression pattern. The DDEGs are listed in Table S4.

### Gene Ontology (GO) analysis

GO analysis of DEGs was performed by Metascape (Zhou et al., 2019) and visualized with the ggplot2 R package and Cytoscape (version 3.7.2). Representative terms selected from the top 100 ranked GO terms or pathways (*P* < 0.01) were displayed.

### Gene set variation analysis

To comprehensively assess the expression levels of individual gene in the pathways, we also adopted Gene set variation analysis (GSVA) (Hanzelmann et al., 2013), a quantitative gene set enrichment analysis method suitable for single-cell transcriptome data, to assign pathway activity estimates of individual cells. GSVA was performed using the GSVA R package (version 1.34.0). The screened signaling pathway gene sets were downloaded from the MsigDB database. The gene-by-cell matrix was converted to gene-set-by-cell matrix and GSVA scores were computed for gene sets with a minimum of five detected genes. The R package limma (version 3.42.2) (Ritchie et al., 2015) was used to calculate significantly enriched pathways. Only pathways that passed two-sided unpaired *t*-tests and Benjamini-Hochberg multiple testing p.adjust were used for downstream analysis.

### Transcriptional regulatory network analysis

The transcriptional regulatory network analysis was performed with the GENIE3 (version 1.6.0) (Huynh-Thu et al., 2010) and RcisTarget (version 1.4.1) R packages of the SCENIC (version 1.1.2.2) (Aibar et al., 2017) workflow using default parameters. Transcription factors (TFs) of mm10 were used as reference TFs and downloaded using RcisTarget. Co-expression modules were first identified between TFs and the potential target genes using the gene expression matrix through GENIE3. Next, for each co-expression module, the cis-regulatory motif enrichment analysis was carried out among all potential target genes using RcisTarget, and only the target genes enriched with the motifs of the corresponding TFs were selected as direct target genes. Each transcription factor and its direct target genes were defined as a regulon. Aging-related gene regulatory networks were inferred. Finally, visualization of TF module networks was performed by Cytoscape (version 3.7.2).

### Gene set score analysis

To score individual cells for pathway activities, we used the R package AUCell (version 1.8.0) (Aibar et al., 2017). We used an expression matrix to compute gene expression rankings in each cell with the “*AUCell_buildRankings*” function, with default parameters. The canonical pathway database was downloaded from the MsigDB database, KEGG database and GO database. These gene sets were used to score each cell. Area-under-the-curve (AUC) values were calculated (“*AUCell_calcAUC*” function) based on gene expression rankings. Genes in each gene set are listed in Table S3.

### RNA-seq library construction and sequencing

Mouse SV cells were transduced with the sg-NTC (Control), sg-*Hsp90aa1* sgRNA were collected for RNA-seq analysis using an Illumina sequencing platform. RNA sequencing libraries were prepared as previously reported (Podnar et al., 2014; Bi et al., 2020; Wang et al., 2022). Briefly, total RNA was extracted using TRIzol™ Reagent (Thermo Fisher Scientific) and RNA integrity was examined by the Bioanalyzer 2100 system (Agilent Technologies). Sequencing libraries were constructed using NEBNext UltraTM RNA Library Prep Kit for Illumina (NEB) and sequenced on Illumina Hiseq X Ten platform. Duplicates were used for the RNA-seq data for each cell type to eliminate technical noise.

### RNA-seq data analysis

Trim Galore (version 0.4.5) software was used for automate adapter trimming and quality control, and Hisat2 (version 2.0.4) (Kim et al., 2015) with default parameters was used to map the cleaned reads to the UCSC mm10 mouse genome. HTSeq (version 0.6.1) (Anders et al., 2015) software was used to count the number of reads mapped in each annotated gene based on the mapping results. R package DESeq2 (version 1.2.4) (Love et al., 2014) was used to calculate DEGs with the cutoff values of Benjamini-Hochberg adjusted *P-*value (p.adjust) < 0.05 and |log_2_ (fold change)| > 0.58. The DEGs are listed in Table S5.

### Statistical analysis

Data are presented as the mean ± SEMs. The statistical analyses were performed using GraphPad Prism (v8) with unpaired two tailed Student’s *t*-test. For gene set score analysis, statistical analysis was performed using two-sided Wilcoxon rank-sum tests. *P*-values < 0.05 are considered statistically significant.

## Supplementary data

The online version contains supplementary material available at https://doi.org/10.1093/procel/pwac058.

pwac058_suppl_Supplementary_Figures

pwac058_suppl_Supplementary_Table_S1

pwac058_suppl_Supplementary_Table_S2

pwac058_suppl_Supplementary_Table_S3

pwac058_suppl_Supplementary_Table_S4

pwac058_suppl_Supplementary_Table_S5

## Data Availability

The raw sequence data reported in this article have been deposited in the Genome Sequence Archive in National Genomics Data Center, China National Center for Bioinformation/Beijing Institute of Genomics, Chinese Academy of Sciences (GSA: CRA004814). The data can also be accessed via an interactive user-friendly webtool at Aging Atlas (Aging Atlas, 2021).

## Abbreviations

ABRs: auditory brainstem responses
ARHL: age-related hearing loss
BC: basal cell
CV: coefficient of variation
DDEGs: dynamic differentially expressed genes
DEGs: differentially expressed genes
DPOAEs: distortion product otoacoustic emissions
ER: endoplasmic reticulum
GO: Gene Ontology
HC: hair cell
H&E: hematoxylin and eosin
IC: intermediate cell
MC: marginal cell
PCA: principal component analysis
PCs: principal components
PDEGs: pairwise differentially expressed genes
RT: room temperature
SC: Schwann cell
scRNA-seq: single-cell RNA sequencing
SGC: satellite glial cell
SGN: spiral ganglion neuron
TF: transcription factor
TUNEL: terminal deoxynucleotidyl transferase dUTP nick end labeling
UMAP: Uniform manifold approximation and projection
UPR: unfolded protein response
