# Supplementary material for: Single-cell transcriptomic atlas of mouse cochlear aging
Source: Protein Cell. 2022 Nov 11;14(3):180–201. doi: 10.1093/procel/pwac058 (PMC10098046; doi:10.1093/procel/pwac058)
Supplement: pwac058_suppl_Supplementary_Figures [file pwac058_suppl_supplementary_figures.pdf]

Supplemental Materials

Figure S1. Aging-associated phenotypic analysis of mouse cochlea.

(A) Statistical data of relative number of outer hair cells in 1-, 5-, and 15-month-old mice. Data are shown as mean ± SEMs (*n* = 5 mice). Two-tailed student's *t*-test *P*-values were indicated. Month, M.

(B) Immunofluorescence analysis of Myo7a in the 1-, 5-, and 15-month-old cochlear samples. Scale bars, 20 μm. The relative number of Myo7a-positive cells is quantified as fold changes and presented as the mean ± SEMs (*n* = 5 mice). Two-tailed student's *t*-test *P*-values were indicated.

(C) Examination of cochlear basilar membranes under the scanning electron microscope in the 1-, 10-, and 20-month-old cochlear samples. Scale bars, 100 μm. The relative numbers of OHCs and IHCs are quantified as fold changes and presented as the mean ± SEMs (*n* = 3 mice). Two-tailed student's *t*-test *P*-values were indicated. The red arrowheads indicate the loss of IHCs, and the yellow ones indicate the loss of OHCs.

(D) Statistical data of relative cell density in cochlear modiolus from 1-, 5-, and 15-month-old mice. Data are shown as mean ± SEMs (*n* = 5 mice). Two-tailed student's *t*-test *P*-values were indicated. Month, M.

(E) Immunofluorescence staining of TUJ1 in the 1-, 5-, and 15-month-old cochlear samples. The relative TUJ1-positive cells at the 1-, 5-, and 15-month-old mice cochleae are quantified as fold changes and presented as the mean ± SEMs (*n* = 5 mice). Two-tailed student's *t*-test *P*-values were indicated. Month, M. Scale bars, 80 μm and 5 μm (zoomed-in images).

(F) Statistical data of relative thickness of stria vascularis at the 1-, 5-, and 15-month-old mice cochleae. Data are shown as mean ± SEMs (*n* = 5 mice). Two-tailed student's *t*-test *P*-values were indicated. Month, M.

(G) Statistical data of relative density of cells in stria vascularis in the 1-, 5-, and 15-month-old mice cochleae. Data are shown as mean ± SEMs (*n* = 5 mice). Two-tailed student's *t*-test *P*-values were indicated. Month, M.

(H) Statistical data of relative density of cells in spiral ligament in the 1-, 5-, and 15-month-old mice cochleae. Data are shown as mean ± SEMs (*n* = 5 mice). Two-tailed student's *t*-test *P*-values were indicated. Month, M.

(I) Statistical data of relative 4-HNE fluorescence intensity at the 1-, 5-, and 15-month-old mice cochleae. Data are shown as mean ± SEMs (*n* = 5 mice). Two-tailed student's *t*-test *P*-values were indicated. Month, M.

(J) Immunofluorescence staining showed neutrophils located in the cochlear spiral ligament. Scale bars, 40 μm and 10 μm (zoomed-in images). The relative percentage is quantified as fold changes relative to that of apical turn in 1-month-old cochlea (*n* = 5 mice). Two-tailed student's *t*-test *P*-values were indicated.

Figure S2. Construction of single-cell atlas of mouse cochlear aging.

(A) Bar plot showing the distribution of quality control for each sample. Reads mapping rate represents the fraction of reads mapped to the genome.

(B) The plot showing the proportion of mitochondrial genes.

(C) The density plot showing the distribution of the number of genes per cell.
(D) The density plot showing the distribution of number of UMI per cell.
(E) UMAP plots showing the cochlear cell distribution from various ages. Month, M.
(F) Principal component analysis (PCA) of cochlear single-cell transcriptome data from each age group. The age and sex of each sample are annotated on the dot. M, Male. F, Female.
(G) Representative GO terms of PDEGs of the male (left) and female (right) across four pairwise comparisons between different age groups.

Figure S3. Cochlear cell subpopulation analysis.
(A) Dot plot showing the expression of canonical cell-type-specific marker genes for diverse cell types.
(B) Boxplot showing the value of ROGUE of each cell type. Box shows the median and the quartile range (25-75%) and the length of whiskers represents 1.5 × the IQR.
(C) UMAP plot showing the distribution of subclusters of DC_PC.
(D) Violin and box plots showing the gene set scores of DC (top) or PC (bottom) signature genes in different subclusters of DC_PC. Signature scores of DC marker genes are relatively higher in cluster 0, 3, 4, 8, 10, thus combinedly named as DC subtype. Whereas, scores of PC marker genes are higher in cluster 1, 2, 6, 7, 9, which were defined as PC subtype. The remaining cluster 5 co-expressed the markers for DC and PC and was defined as a mixed subtype. Boxes show the medians and the quartile ranges (25-75%) and the lengths of whiskers represent 1.5 × the IQR.
(E) Violin plots showing the expression level of selected marker genes that are differentially expressed in the DC and PC.
(F) UMAP plot showing the distribution of DC_PC subpopulations, including DC, PC, and mixed subtype.
(G) UMAP plot showing the distribution of PC subpopulations.
(H) Left, heatmap showing the expression profile of marker genes of PC and *Nudt4*$^{+}$ PC. Right, bar plot showing the representative GO terms enriched for the marker genes in PC and *Nudt4*$^{+}$ PC.
(I) Left, UMAP plot showing the distribution of IHCs and OHCs. Right, bar plot showing the representative GO terms enriched for the marker genes in IHC and OHC.
(J) Left, UMAP plot showing the distribution of type I and II SGNs. Right, bar plot showing the representative GO terms enriched for the marker genes in type I and II SGNs.

Figure S4. Coefficient of variation analysis of the mouse cochlea across all cell types.
(A) Boxplots showing the coefficient of variation of each cell type from 3 different comparisons including 2 M vs. 1 M (left), 5 M vs. 1 M (middle) and 15 M vs. 1 M (right).
(B) Heatmap showing the row Z-score expression level of genes with high Pearson's correlation coefficients (correlation coefficient > 0.6 and FDR < 0.05) between coefficient of variation and expression levels in intermediate cells. The bins are

arranged based on the coefficient of variation rank in each group.

(C) The scatter plots showing the high correlation between coefficient of variation and average gene expression level in intermediate cells (5 M vs. 1 M).

Figure S5. Pairwise differential expression analysis pinpoints cell type-specific temporal signatures during cochlear aging.

(A) Heatmap showing the frequency of upregulated PDEGs shared by more than 12 cell types.

(B) Heatmap showing the frequency of downregulated PDEGs shared by more than 12 cell types.

(C) Representative GO terms of upregulated PDEGs shared by less than 3 cell types.

(D) Representative GO terms of downregulated PDEGs shared by less than 3 cell types.

Figure S6. Functional enrichment analysis of the pairwise differentially expressed genes.

(A)-(B) Representative GO terms of age-specific upregulated (A) or downregulated (B) PDEGs. Count indicates gene number.

Figure S7. Gene expression signatures for the pairwise differentially expressed genes.

(A)-(B) GSVA analysis of pathway enrichment of upregulated (A) and downregulated (B) PDEGs across four pairwise comparisons between different age groups.

(C) Network visualization of upregulated and downregulated core regulatory transcription factors in four different comparisons of all cell types. Node size positively correlates with the number of target genes.

(D) Dot plots showing the PDEGs overlapped with genes from Aging Atlas database. The node size indicates the frequency of PDEGs appearing across four pairwise comparisons.

Figure S8. GSVA analysis of dynamic differentially expressed genes.

(A) GSVA analysis of pathway enrichment of upregulated (left) and downregulated (right) DDEGs across four pairwise comparisons between different age groups. The size of each bar indicates the t-value for each pathway (two-sided unpaired limma-moderated t-test).

(B) Violin and box plots showing the GSVA scores (scored per cell by GSVA) of upregulated (top) and downregulated (bottom) DDEGs related to key pathways in cochlear cells across different timepoints.

Figure S9. Gene expression signatures of dynamic differentially expressed genes and the upregulation of *Hsp90aa1* expression in aged cochlear tissue.

(A) Left, heatmap showing the relative expression levels of *Calr* in cochlear cells from 1-, 2-, 5-, 12-, and 15-month-old mice. Right, immunofluorescence analysis of CALR in the 1-, 5-, and 15-month-old cochlear samples. The pink and white boxes indicate the magnified regions of stria vascularis and spiral ligament, respectively. Scale bars,

20 μm. The relative fluorescence intensity was quantified as fold changes and presented as the mean ± SEMs (*n* = 5 mice). Two-tailed student's *t*-test *P*-values were indicated.

(B) Left, heatmap showing the relative expression levels of *S100a8* in cochlear cells from 1-, 2-, 5-, 12-, and 15-month-old mice. Right, immunofluorescence analysis of S100A8 in the 1-, 5-, and 15-month-old cochlear samples. Scale bars, 20 μm. The relative fluorescence intensity was quantified as fold changes and presented as the mean ± SEMs (*n* = 5 mice). Two-tailed student's *t*-test *P*-values were indicated.

(C) Left, heatmap showing the relative expression levels of *S100a9* in cochlear cells from 1-, 2-, 5-, 12-, and 15-month-old mice. Right, immunofluorescence analysis of S100A9 in the 1-, 5-, and 15-month-old cochlear samples. Scale bars, 20 μm. The relative fluorescence intensity was quantified as fold changes and presented as the mean ± SEMs (*n* = 5 mice). Two-tailed student's *t*-test *P*-values were indicated.

(D)-(E) Representative GO terms of shared upregulated (d) or downregulated (e) DDEGs. Counts indicate gene number.

(F) Heatmap showing the relative expression levels of upregulated (left) and downregulated (right) DDEGs in HCs.

(G) Heatmap showing the relative expression levels of upregulated DDEGs in SGNs.

Figure S10. Transcriptional profiles for the activation of HSP90AA1 in stria vascularis cells.

(A) Network visualization of upregulated and downregulated core regulatory transcription factors in intermediate cells. Node size positively correlates with the number of target genes. *Hsp90aa1* was highlighted in yellow.

(B) Western blotting and band intensity quantification of HSP90AA1 protein levels in cochlear tissues from young and aged mice. The relative protein level was quantified as fold changes and presented as the mean ± SEMs (*n* = 5 mice). Two-tailed student's *t*-test *P*-value was indicated.

(C) Western blotting showing the protein level of KCNJ13 in OP9 and SV-k1 cell lines. β-Tubulin was used as loading control. The protein levels are quantified as fold changes and presented as mean ± SEMs (*n* = 6). Two-tailed student's *t*-test *P*-value was indicated.

(D) Principal component analysis (PCA) of bulk transcription data from each group. Each sample is annotated on the dot.

(E) Volcano plot showing the DEGs between sg-NTC (TM) and sg-NTC (Vehicle).

(F) Heatmap showing the row z-score expression of DEGs between sg-NTC (TM) and sg-NTC (Vehicle) and corresponding representative GO terms. Count indicates gene number.

(G) Western blotting and band intensity quantification of HSP90AA1 protein levels in SV-k1 cells transduced with non-targeting or *Hsp90aa1*-targeting sgRNA. Data are presented as the mean ± SEMs, *n* = 3 biological repeats. Two-tailed student's *t*-test *P*-values were indicated. A representative data from one of the two independent experiments. sg-N, sg-NC; sg-H, sg-*Hsp90aa1*.

(H) Left, aggresome intensity analysis of stria vascularis cells transduced with non-targeting or *Hsp90aa1*-targeting sgRNA after treatment with vehicle (Veh) or TM.

Right, data are presented as mean ± SEMs, $n = 3$ biological repeats. Two-tailed student's t-test P-values were indicated. A representative data from one of the two independent experiments.

(I) Left, apoptosis analysis of stria vascularis cells transduced with non-targeting or *Hsp90aa1*-targeting sgRNA after treatment with vehicle or TM. Right, the percentages of apoptotic cells are presented as mean ± SEMs, $n = 3$ biological repeats. Two-tailed student's t-test P-values were indicated. A representative data from one of the two independent experiments.

(J) Volcano plot showing the DEGs between sg-*Hsp90aa1* (TM) and sg-NTC (TM).

(K) Heatmap showing the row Z-score expression of DEGs between sg-*Hsp90aa1* (TM) and sg-NTC (TM) and corresponding representative GO terms. Count indicates gene number.

Supplementary Table Legends

Table S1. Marker genes for each cell type in mouse cochlea.
Table S2. Pairwise differentially expressed genes (PDEGs) for each cell type.
Table S3. List of age-related genes in different database or pathways used in this study.
Table S4. Age-dependent dynamic differentially expressed genes (DDEGs) for each cell type.
Table S5. Differentially expressed genes (DEGs) in bulk RNA-seq data.

Figure S1

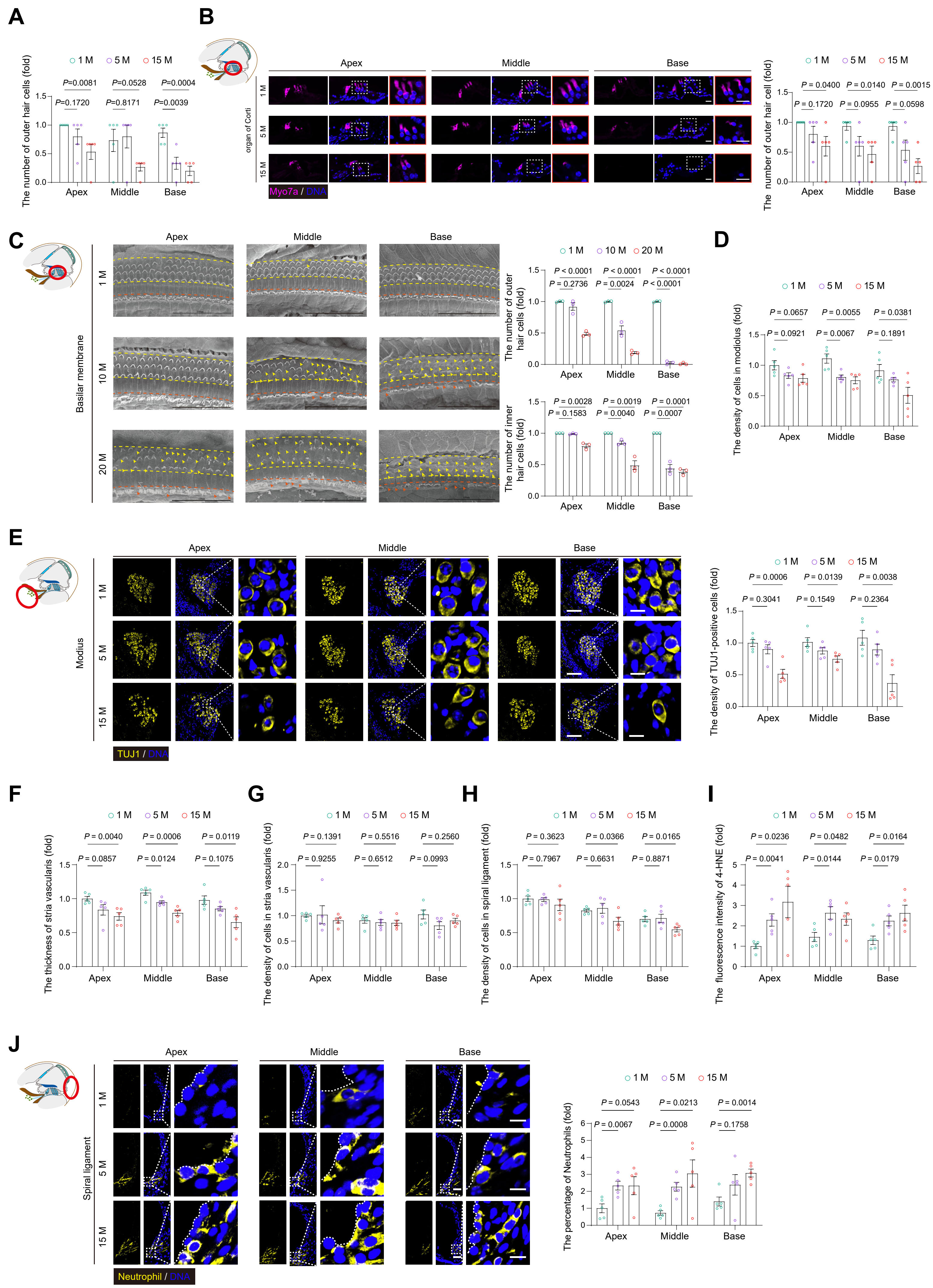

A
1 M
5 M
15 M
The number of outer hair cells (fold)
P=0.0081
P=0.0528
P=0.0004
P=0.1720
P=0.8171
P=0.0039
Apex
Middle
Base
B
Apex
Middle
Base
organ of Corti
1 M
5 M
15 M
Myo7a / DNA
The number of outer hair cell (fold)
P = 0.0400
P = 0.0140
P = 0.0015
P = 0.1720
P = 0.0955
P = 0.0598
C
Apex
Middle
Base
Basilar membrane
1 M
10 M
20 M
The number of outer hair cells (fold)
P < 0.0001
P < 0.0001
P < 0.0001
P = 0.2736
P = 0.0024
P < 0.0001
The number of inner hair cells (fold)
P = 0.0028
P = 0.0019
P = 0.0001
P = 0.1583
P = 0.0040
P = 0.0007
D
The density of cells in modiolus (fold)
P = 0.0657
P = 0.0055
P = 0.0381
P = 0.0921
P = 0.0067
P = 0.1891
E
Modius
TUJ1 / DNA
The density of TUJ1-positive cells (fold)
P = 0.0006
P = 0.0139
P = 0.0038
P = 0.3041
P = 0.1549
P = 0.2364
F
The thickness of stria vascularis (fold)
P = 0.0040
P = 0.0006
P = 0.0119
P = 0.0857
P = 0.0124
P = 0.1075
G
The density of cells in stria vascularis (fold)
P = 0.1391
P = 0.5516
P = 0.2560
P = 0.9255
P = 0.6512
P = 0.0993
H
The density of cells in spiral ligament (fold)
P = 0.3623
P = 0.0366
P = 0.0165
P = 0.7967
P = 0.6631
P = 0.8871
I
The fluorescence intensity of 4-HNE (fold)
P = 0.0236
P = 0.0482
P = 0.0164
P = 0.0041
P = 0.0144
P = 0.0179
J
Spiral ligament
Neutrophil / DNA
The percentage of Neutrophils (fold)
P = 0.0543
P = 0.0213
P = 0.0014
P = 0.0067
P = 0.0008
P = 0.1758

Figure S2

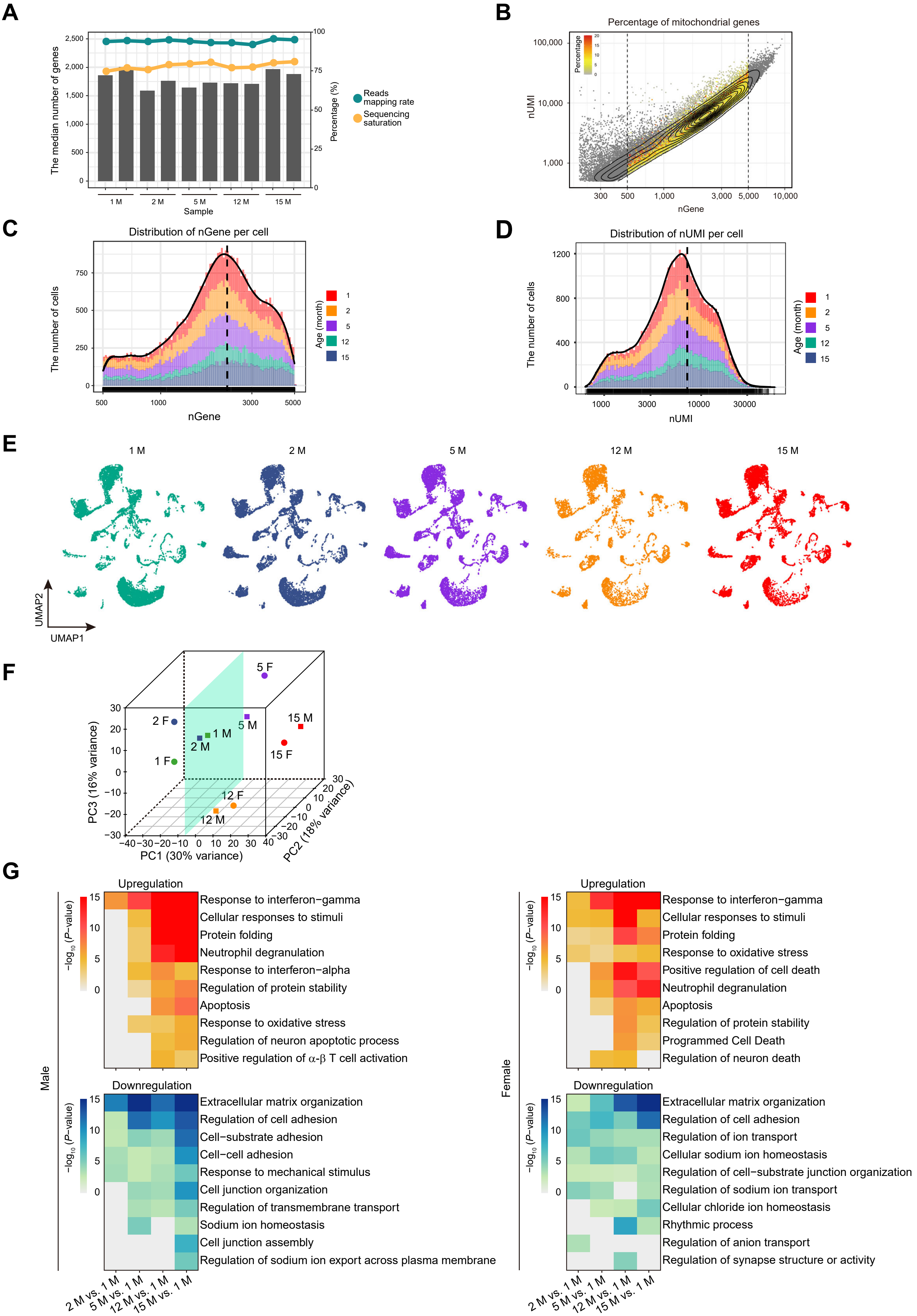
A
The median number of genes
Percentage (%)
Reads mapping rate
Sequencing saturation
1 M
2 M
5 M
12 M
15 M
Sample
B
Percentage of mitochondrial genes
Percentage
nUMI
nGene
C
Distribution of nGene per cell
The number of cells
nGene
Age (month)
D
Distribution of nUMI per cell
The number of cells
nUMI
Age (month)
E
1 M
2 M
5 M
12 M
15 M
UMAP1
UMAP2
F
PC1 (30% variance)
PC2 (18% variance)
PC3 (16% variance)
1 F
2 F
5 F
12 F
15 F
1 M
2 M
5 M
12 M
15 M
G
Male
Female
Upregulation
Response to interferon−gamma
Cellular responses to stimuli
Protein folding
Neutrophil degranulation
Response to interferon−alpha
Regulation of protein stability
Apoptosis
Response to oxidative stress
Regulation of neuron apoptotic process
Positive regulation of α-β T cell activation
Downregulation
Extracellular matrix organization
Regulation of cell adhesion
Cell−substrate adhesion
Cell−cell adhesion
Response to mechanical stimulus
Cell junction organization
Regulation of transmembrane transport
Sodium ion homeostasis
Cell junction assembly
Regulation of sodium ion export across plasma membrane
Upregulation
Response to interferon−gamma
Cellular responses to stimuli
Protein folding
Response to oxidative stress
Positive regulation of cell death
Neutrophil degranulation
Apoptosis
Regulation of protein stability
Programmed Cell Death
Regulation of neuron death
Downregulation
Extracellular matrix organization
Regulation of cell adhesion
Regulation of ion transport
Cellular sodium ion homeostasis
Regulation of cell−substrate junction organization
Regulation of sodium ion transport
Cellular chloride ion homeostasis
Rhythmic process
Regulation of anion transport
Regulation of synapse structure or activity
−log10 (P−value)
2 M vs. 1 M
5 M vs. 1 M
12 M vs. 1 M
15 M vs. 1 M

Figure S3

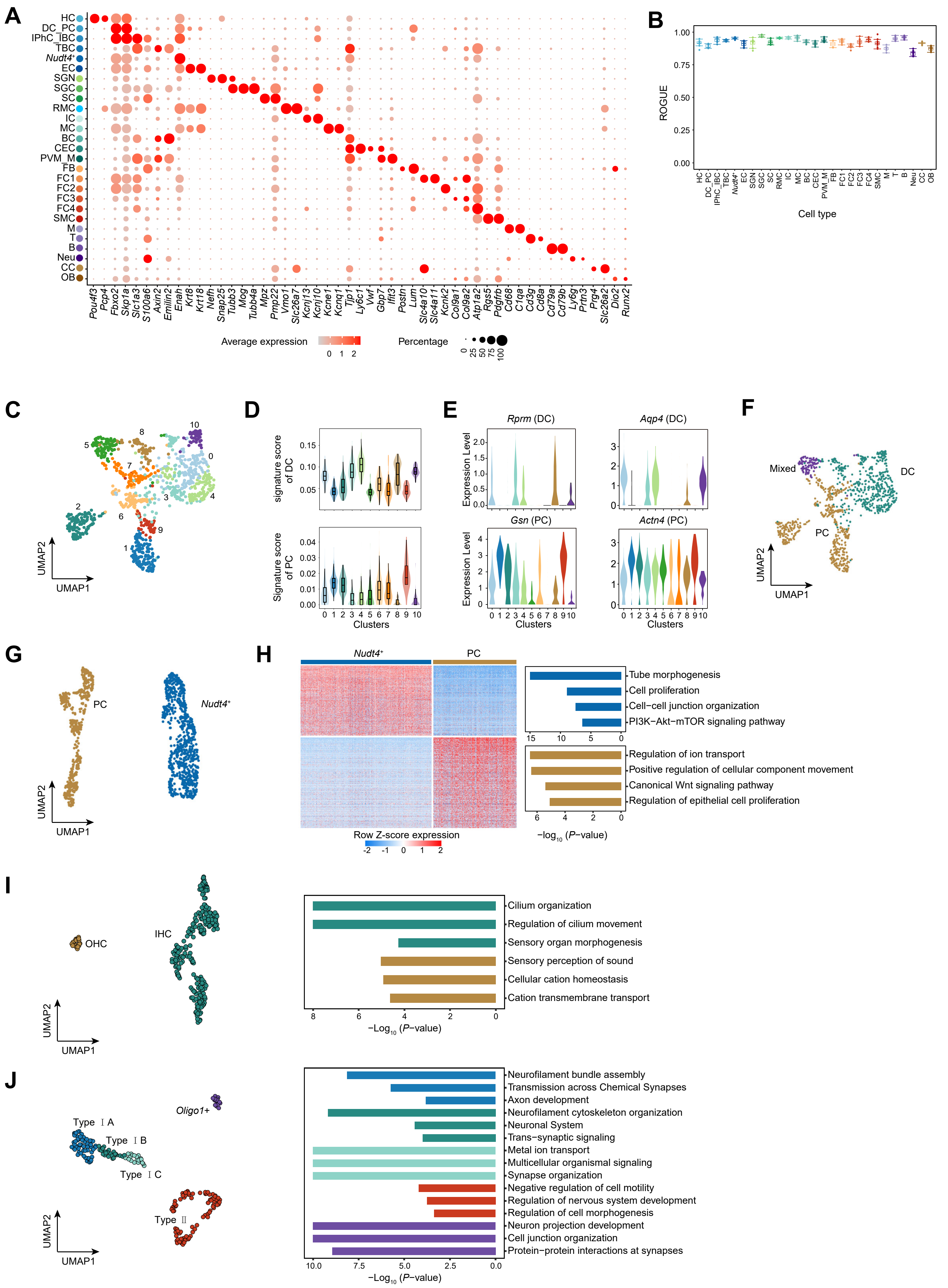

A
B
ROGUE
Cell type
Average expression
Percentage
C
D
E
F
G
H
I
J
UMAP1
UMAP2
Clusters
Expression Level
signature score of DC
Signature score of PC
Rprm (DC)
Aqp4 (DC)
Gsn (PC)
Actn4 (PC)
Mixed
DC
PC
Nudt4+
Row Z-score expression
Tube morphogenesis
Cell proliferation
Cell−cell junction organization
PI3K−Akt−mTOR signaling pathway
Regulation of ion transport
Positive regulation of cellular component movement
Canonical Wnt signaling pathway
Regulation of epithelial cell proliferation
−log10 (P-value)
OHC
IHC
Cilium organization
Regulation of cilium movement
Sensory organ morphogenesis
Sensory perception of sound
Cellular cation homeostasis
Cation transmembrane transport
−Log10 (P-value)
Oligo1+
Type Ⅰ A
Type Ⅰ B
Type Ⅰ C
Type Ⅱ
Neurofilament bundle assembly
Transmission across Chemical Synapses
Axon development
Neurofilament cytoskeleton organization
Neuronal System
Trans−synaptic signaling
Metal ion transport
Multicellular organismal signaling
Synapse organization
Negative regulation of cell motility
Regulation of nervous system development
Regulation of cell morphogenesis
Neuron projection development
Cell junction organization
Protein−protein interactions at synapses

Figure S4

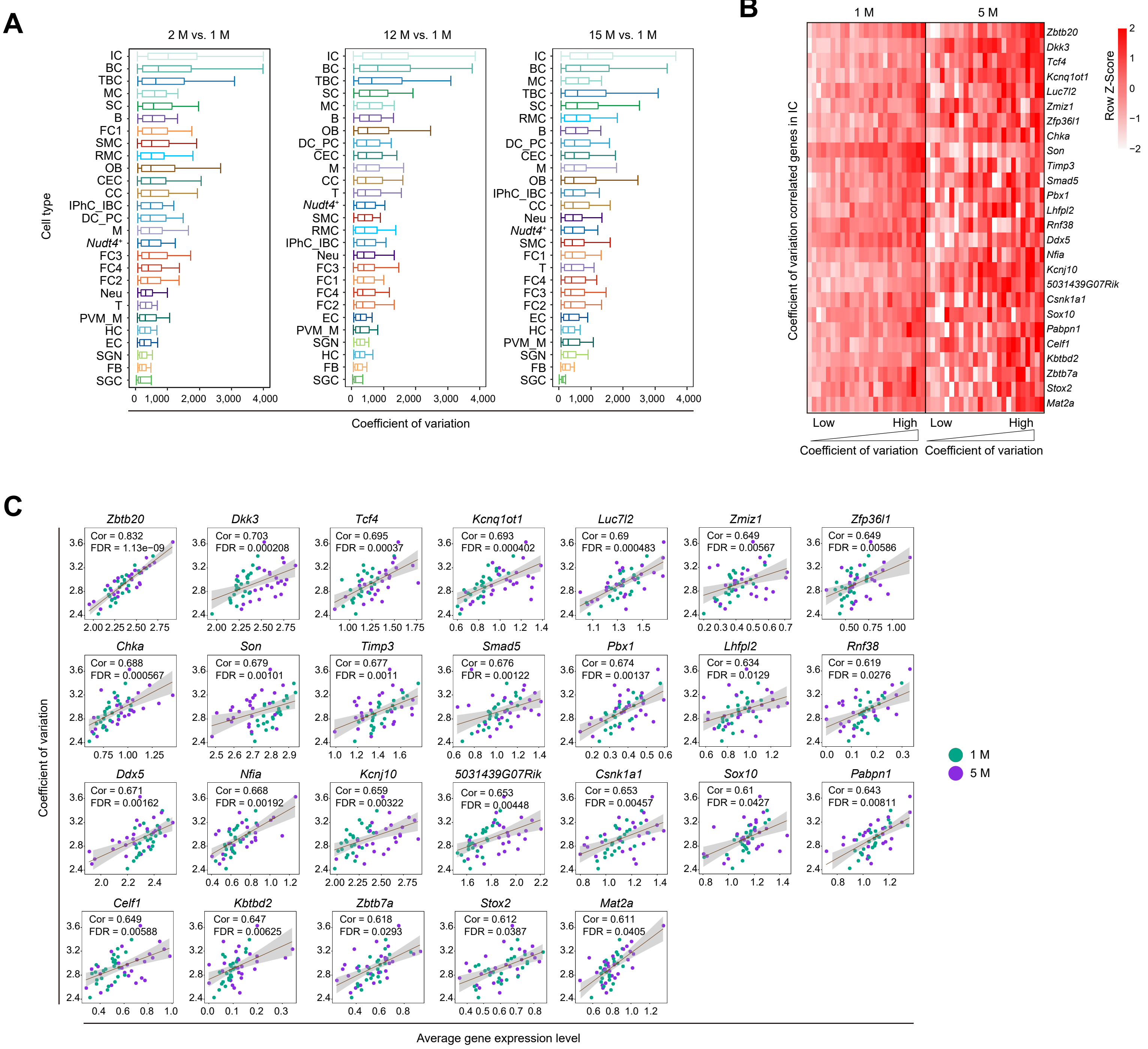
A
2 M vs. 1 M
12 M vs. 1 M
15 M vs. 1 M
Cell type
Coefficient of variation
B
1 M
5 M
Row Z-Score
Coefficient of variation correlated genes in IC
Zbtb20
Dkk3
Tcf4
Kcnq1ot1
Luc7l2
Zmiz1
Zfp36l1
Chka
Son
Timp3
Smad5
Pbx1
Lhfpl2
Rnf38
Ddx5
Nfia
Kcnj10
5031439G07Rik
Csnk1a1
Sox10
Pabpn1
Celf1
Kbtbd2
Zbtb7a
Stox2
Mat2a
Low
High
Coefficient of variation
C
Zbtb20 Cor = 0.832 FDR = 1.13e−09
Dkk3 Cor = 0.703 FDR = 0.000208
Tcf4 Cor = 0.695 FDR = 0.00037
Kcnq1ot1 Cor = 0.693 FDR = 0.000402
Luc7l2 Cor = 0.69 FDR = 0.000483
Zmiz1 Cor = 0.649 FDR = 0.00567
Zfp36l1 Cor = 0.649 FDR = 0.00586
Chka Cor = 0.688 FDR = 0.000567
Son Cor = 0.679 FDR = 0.00101
Timp3 Cor = 0.677 FDR = 0.0011
Smad5 Cor = 0.676 FDR = 0.00122
Pbx1 Cor = 0.674 FDR = 0.00137
Lhfpl2 Cor = 0.634 FDR = 0.0129
Rnf38 Cor = 0.619 FDR = 0.0276
Ddx5 Cor = 0.671 FDR = 0.00162
Nfia Cor = 0.668 FDR = 0.00192
Kcnj10 Cor = 0.659 FDR = 0.00322
5031439G07Rik Cor = 0.653 FDR = 0.00448
Csnk1a1 Cor = 0.653 FDR = 0.00457
Sox10 Cor = 0.61 FDR = 0.0427
Pabpn1 Cor = 0.643 FDR = 0.00811
Celf1 Cor = 0.649 FDR = 0.00588
Kbtbd2 Cor = 0.647 FDR = 0.00625
Zbtb7a Cor = 0.618 FDR = 0.0293
Stox2 Cor = 0.612 FDR = 0.0387
Mat2a Cor = 0.611 FDR = 0.0405
1 M
5 M
Coefficient of variation
Average gene expression level

Figure S5

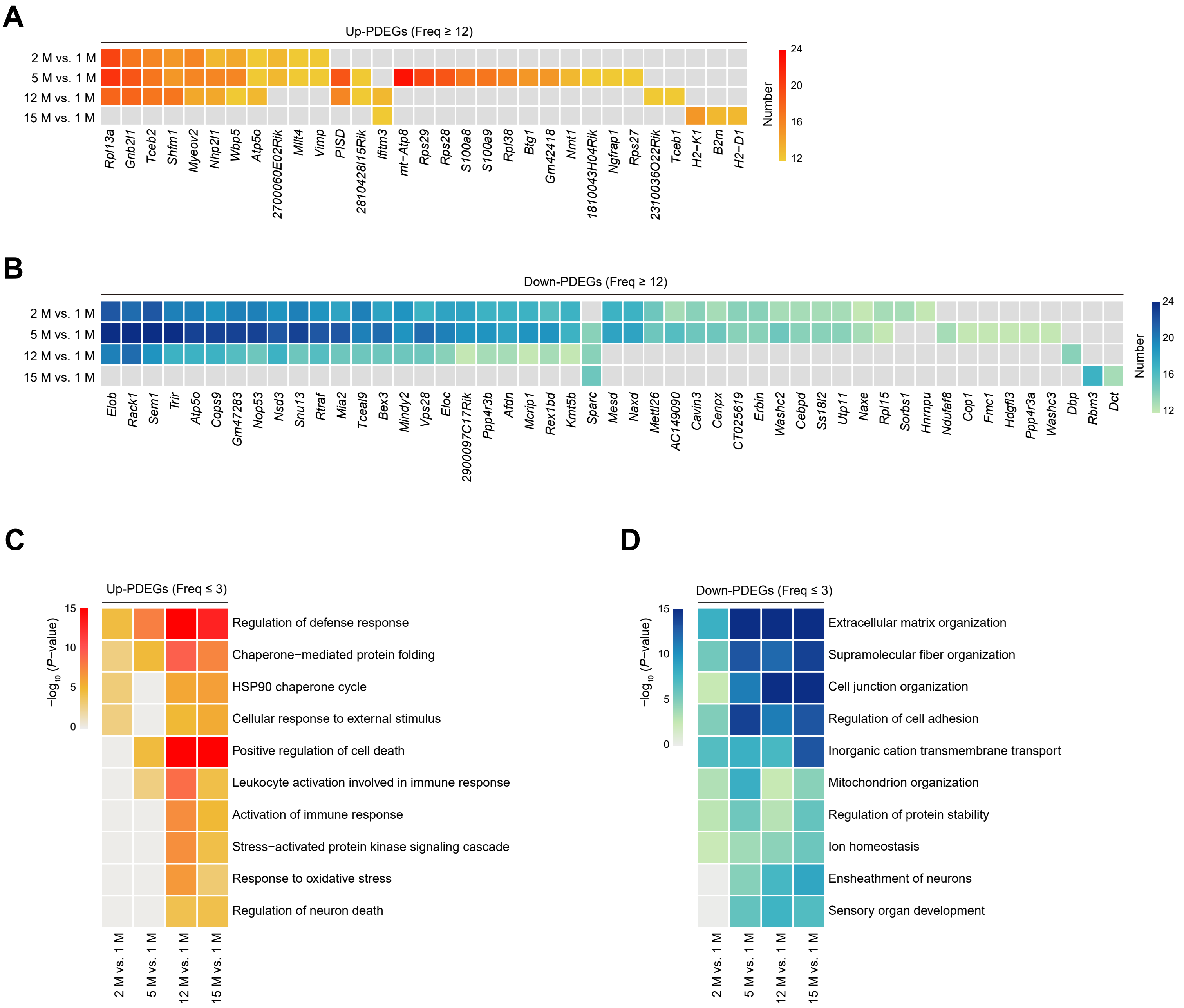
A
Up-PDEGs (Freq ≥ 12)
2 M vs. 1 M
5 M vs. 1 M
12 M vs. 1 M
15 M vs. 1 M
Rpl13a
Gnb2l1
Tceb2
Shfm1
Myeov2
Nhp2l1
Wbp5
Atp5o
2700060E02Rik
Mllt4
Vimp
PISD
2810428I15Rik
Ifitm3
mt−Atp8
Rps29
Rps28
S100a8
S100a9
Rpl38
Btg1
Gm42418
Nmt1
1810043H04Rik
Ngfrap1
Rps27
2310036O22Rik
Tceb1
H2−K1
B2m
H2−D1
Number
24
20
16
12
B
Down-PDEGs (Freq ≥ 12)
2 M vs. 1 M
5 M vs. 1 M
12 M vs. 1 M
15 M vs. 1 M
Elob
Rack1
Sem1
Trir
Atp5o
Cops9
Gm47283
Nop53
Nsd3
Snu13
Rtraf
Mia2
Tceal9
Bex3
Mindy2
Vps28
Eloc
2900097C17Rik
Ppp4r3b
Afdn
Mcrip1
Rex1bd
Kmt5b
Sparc
Mesd
Naxd
Mettl26
AC149090
Cavin3
Cenpx
CT025619
Erbin
Washc2
Cebpd
Ss18l2
Utp11
Naxe
Rpl15
Sorbs1
Hnrnpu
Ndufaf8
Cop1
Fmc1
Hdgfl3
Ppp4r3a
Washc3
Dbp
Rbm3
Dct
Number
24
20
16
12
C
Up-PDEGs (Freq ≤ 3)
−log10 (P−value)
15
10
5
0
Regulation of defense response
Chaperone−mediated protein folding
HSP90 chaperone cycle
Cellular response to external stimulus
Positive regulation of cell death
Leukocyte activation involved in immune response
Activation of immune response
Stress−activated protein kinase signaling cascade
Response to oxidative stress
Regulation of neuron death
2 M vs. 1 M
5 M vs. 1 M
12 M vs. 1 M
15 M vs. 1 M
D
Down-PDEGs (Freq ≤ 3)
−log10 (P−value)
15
10
5
0
Extracellular matrix organization
Supramolecular fiber organization
Cell junction organization
Regulation of cell adhesion
Inorganic cation transmembrane transport
Mitochondrion organization
Regulation of protein stability
Ion homeostasis
Ensheathment of neurons
Sensory organ development
2 M vs. 1 M
5 M vs. 1 M
12 M vs. 1 M
15 M vs. 1 M

Figure S6

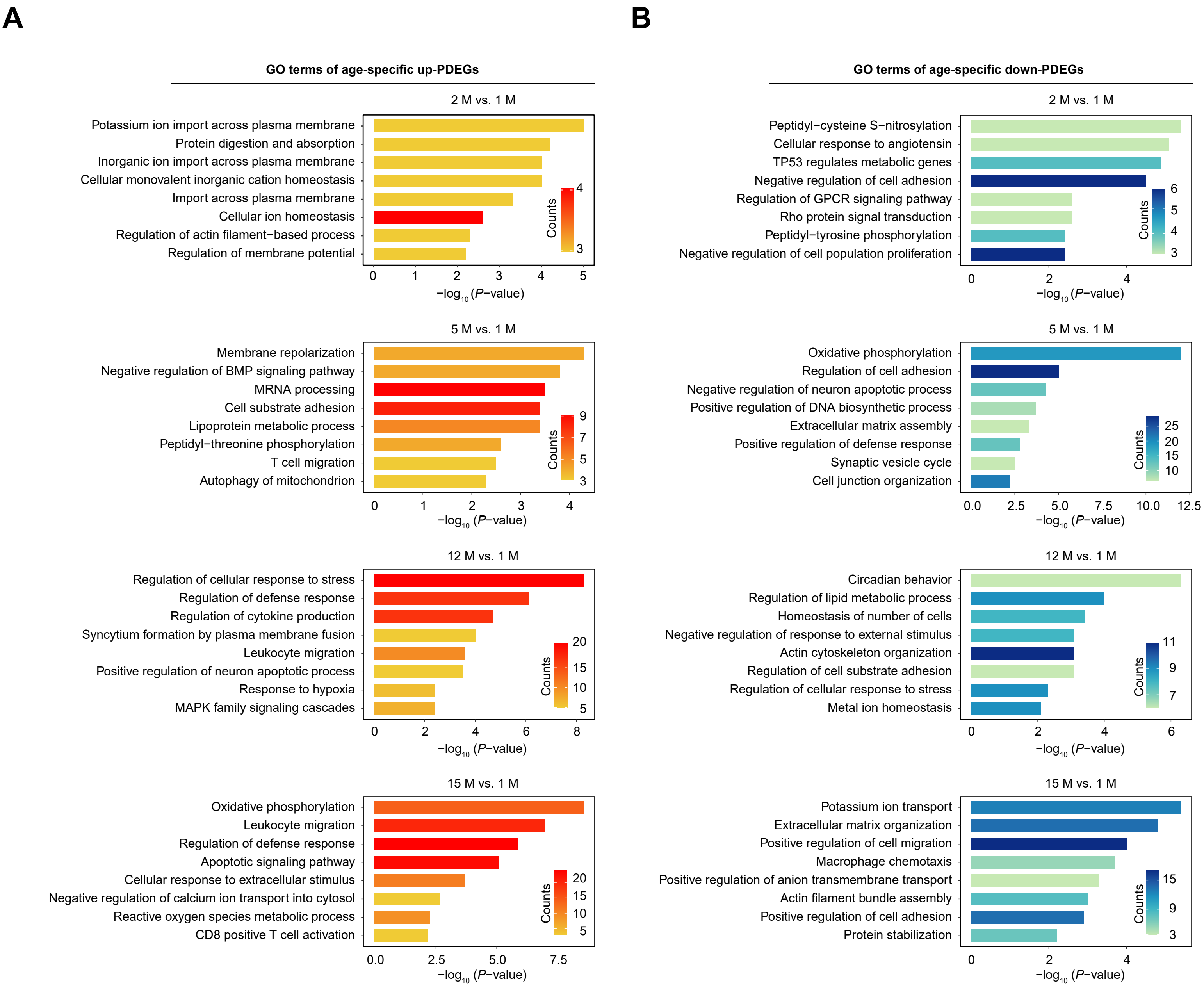
A
B
GO terms of age-specific up-PDEGs
2 M vs. 1 M
Potassium ion import across plasma membrane
Protein digestion and absorption
Inorganic ion import across plasma membrane
Cellular monovalent inorganic cation homeostasis
Import across plasma membrane
Cellular ion homeostasis
Regulation of actin filament−based process
Regulation of membrane potential
Counts
−log10 (P−value)
5 M vs. 1 M
Membrane repolarization
Negative regulation of BMP signaling pathway
MRNA processing
Cell substrate adhesion
Lipoprotein metabolic process
Peptidyl−threonine phosphorylation
T cell migration
Autophagy of mitochondrion
12 M vs. 1 M
Regulation of cellular response to stress
Regulation of defense response
Regulation of cytokine production
Syncytium formation by plasma membrane fusion
Leukocyte migration
Positive regulation of neuron apoptotic process
Response to hypoxia
MAPK family signaling cascades
15 M vs. 1 M
Oxidative phosphorylation
Leukocyte migration
Regulation of defense response
Apoptotic signaling pathway
Cellular response to extracellular stimulus
Negative regulation of calcium ion transport into cytosol
Reactive oxygen species metabolic process
CD8 positive T cell activation
GO terms of age-specific down-PDEGs
2 M vs. 1 M
Peptidyl−cysteine S−nitrosylation
Cellular response to angiotensin
TP53 regulates metabolic genes
Negative regulation of cell adhesion
Regulation of GPCR signaling pathway
Rho protein signal transduction
Peptidyl−tyrosine phosphorylation
Negative regulation of cell population proliferation
5 M vs. 1 M
Oxidative phosphorylation
Regulation of cell adhesion
Negative regulation of neuron apoptotic process
Positive regulation of DNA biosynthetic process
Extracellular matrix assembly
Positive regulation of defense response
Synaptic vesicle cycle
Cell junction organization
12 M vs. 1 M
Circadian behavior
Regulation of lipid metabolic process
Homeostasis of number of cells
Negative regulation of response to external stimulus
Actin cytoskeleton organization
Regulation of cell substrate adhesion
Regulation of cellular response to stress
Metal ion homeostasis
15 M vs. 1 M
Potassium ion transport
Extracellular matrix organization
Positive regulation of cell migration
Macrophage chemotaxis
Positive regulation of anion transmembrane transport
Actin filament bundle assembly
Positive regulation of cell adhesion
Protein stabilization

Figure S7

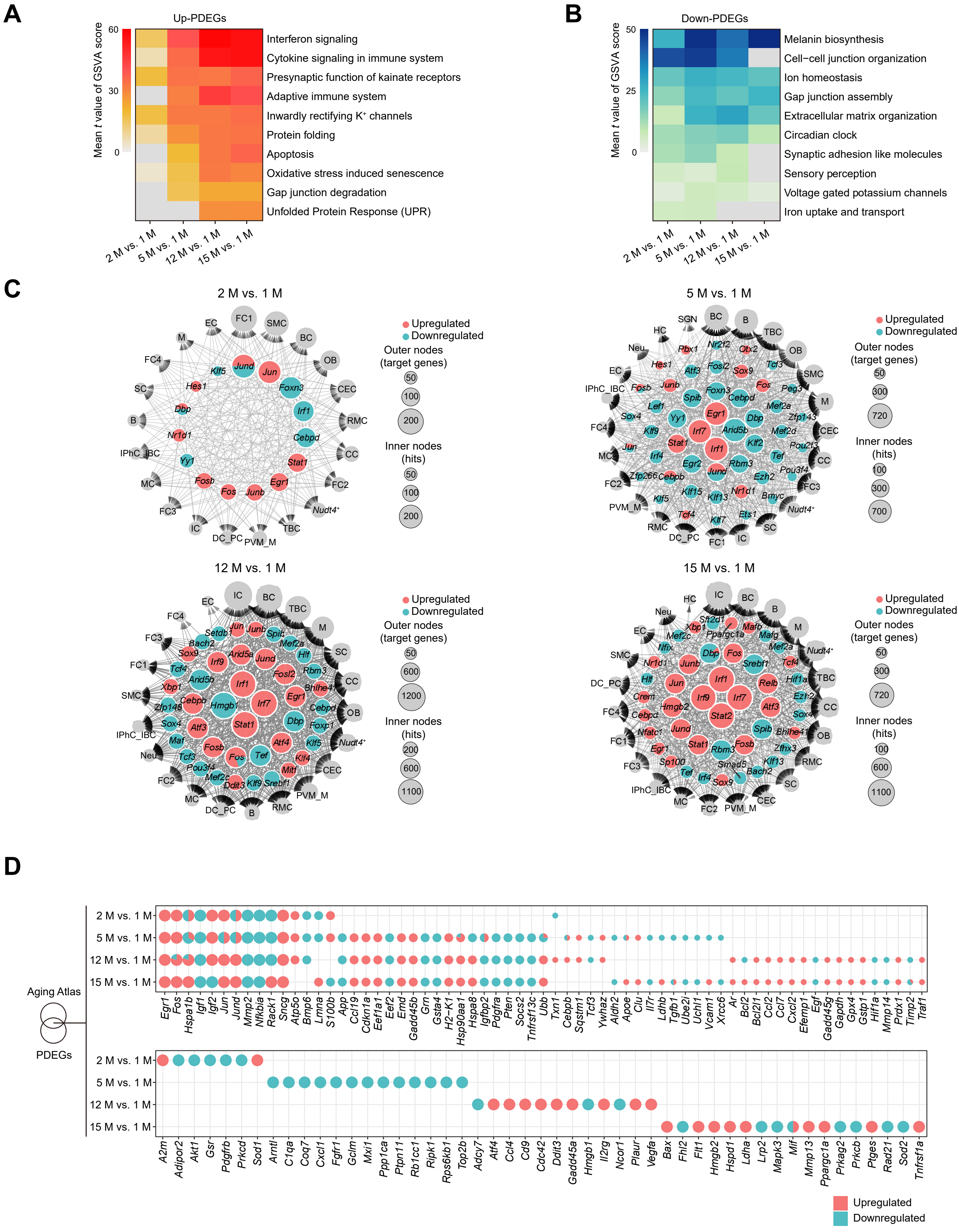
A
Up-PDEGs
Mean t value of GSVA score
60
30
0
Interferon signaling
Cytokine signaling in immune system
Presynaptic function of kainate receptors
Adaptive immune system
Inwardly rectifying K+ channels
Protein folding
Apoptosis
Oxidative stress induced senescence
Gap junction degradation
Unfolded Protein Response (UPR)
2 M vs. 1 M
5 M vs. 1 M
12 M vs. 1 M
15 M vs. 1 M
B
Down-PDEGs
Mean t value of GSVA score
50
25
0
Melanin biosynthesis
Cell−cell junction organization
Ion homeostasis
Gap junction assembly
Extracellular matrix organization
Circadian clock
Synaptic adhesion like molecules
Sensory perception
Voltage gated potassium channels
Iron uptake and transport
2 M vs. 1 M
5 M vs. 1 M
12 M vs. 1 M
15 M vs. 1 M
C
2 M vs. 1 M
5 M vs. 1 M
12 M vs. 1 M
15 M vs. 1 M
Upregulated
Downregulated
Outer nodes (target genes)
Inner nodes (hits)
D
Aging Atlas
PDEGs
2 M vs. 1 M
5 M vs. 1 M
12 M vs. 1 M
15 M vs. 1 M
Egr1 Fos Hspa1b Igf1 Igf2 Jun Jund Mmp2 Nfkbia Rack1 Sncg Atp5o Bmp6 Lmna S100b App Ccl19 Cdkn1a Eef1a1 Eef2 Emd Gadd45b Grn Gsta4 H2−K1 Hsp90aa1 Hspa8 Igfbp2 Pdgfra Pten Socs2 Tnfrsf13c Ubb Txn1 Cebpb Sqstm1 Tcf3 Ywhaz Aldh2 Apoe Clu Il7r Ldhb Tgfb1 Ube2i Uchl1 Vcam1 Xrcc6 Ar Bcl2 Bcl2l1 Ccl2 Ccl7 Cxcl2 Efemp1 Egf Gadd45g Gapdh Gpx4 Gstp1 Hif1a Mmp14 Prdx1 Timp2 Traf1
A2m Adipor2 Akt1 Gsr Pdgfrb Prkcd Sod1 Arntl C1qa Coq7 Cxcl1 Fgfr1 Gclm Mxi1 Ppp1ca Ptpn11 Rb1cc1 Ripk1 Rps6kb1 Top2b Adcy7 Atf4 Ccl4 Cd9 Cdc42 Ddit3 Gadd45a Hmgb1 Il2rg Ncor1 Plaur Vegfa Bax Fhl2 Flt1 Hmgb2 Hspd1 Ldha Lrp2 Mapk3 Mif Mmp13 Ppargc1a Prkag2 Prkcb Ptges Rad21 Sod2 Tnfrsf1a
Upregulated
Downregulated

Figure S8

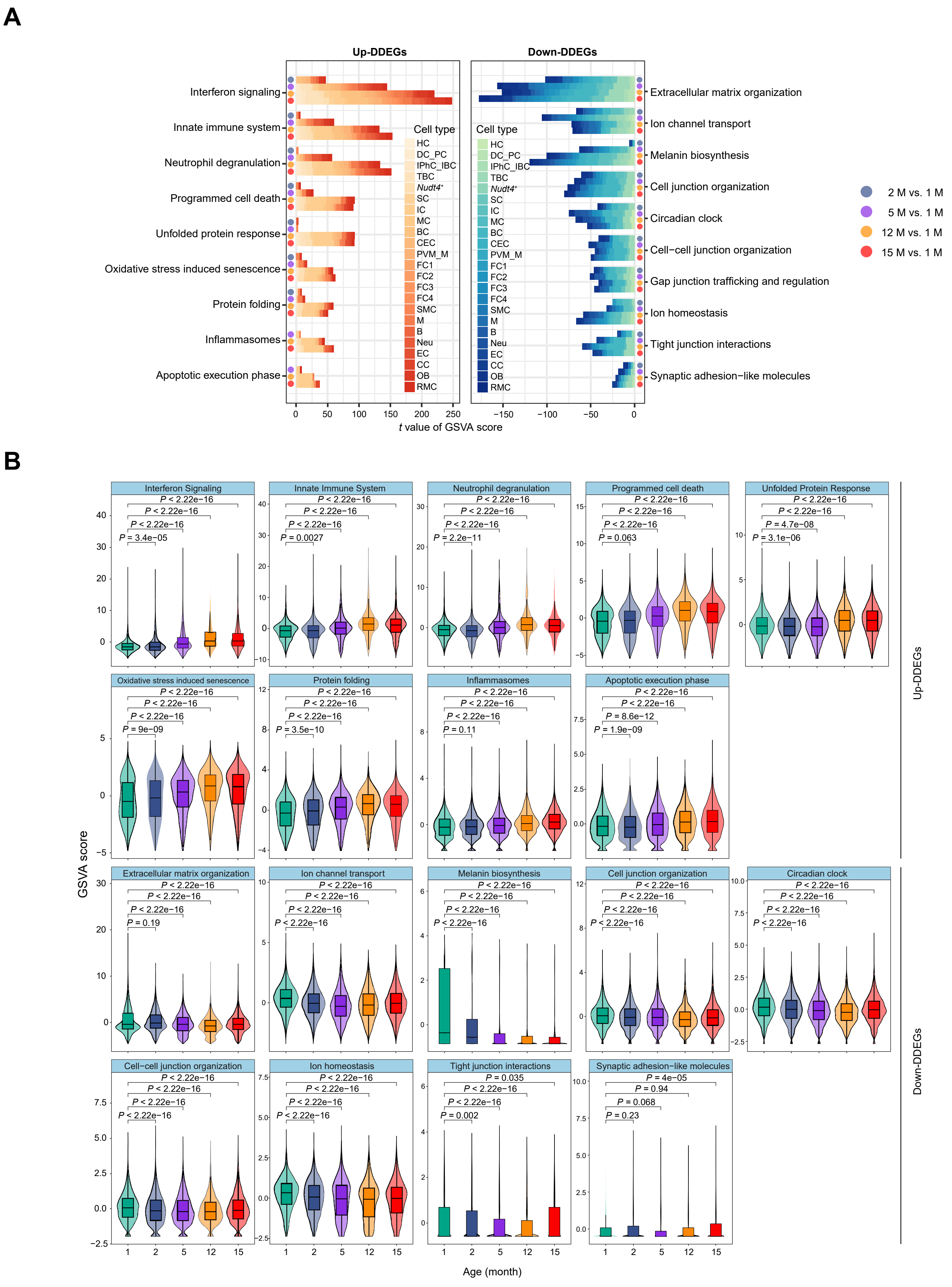
A
Up-DDEGs
Down-DDEGs
Interferon signaling
Innate immune system
Neutrophil degranulation
Programmed cell death
Unfolded protein response
Oxidative stress induced senescence
Protein folding
Inflammasomes
Apoptotic execution phase
Extracellular matrix organization
Ion channel transport
Melanin biosynthesis
Cell junction organization
Circadian clock
Cell−cell junction organization
Gap junction trafficking and regulation
Ion homeostasis
Tight junction interactions
Synaptic adhesion−like molecules
Cell type
HC
DC_PC
IPhC_IBC
TBC
Nudt4+
SC
IC
MC
BC
CEC
PVM_M
FC1
FC2
FC3
FC4
SMC
M
B
Neu
EC
CC
OB
RMC
2 M vs. 1 M
5 M vs. 1 M
12 M vs. 1 M
15 M vs. 1 M
t value of GSVA score
B
Interferon Signaling
Innate Immune System
Neutrophil degranulation
Programmed cell death
Unfolded Protein Response
Oxidative stress induced senescence
Protein folding
Inflammasomes
Apoptotic execution phase
Extracellular matrix organization
Ion channel transport
Melanin biosynthesis
Cell junction organization
Circadian clock
Cell−cell junction organization
Ion homeostasis
Tight junction interactions
Synaptic adhesion−like molecules
GSVA score
Age (month)
Up-DDEGs
Down-DDEGs

Figure S9

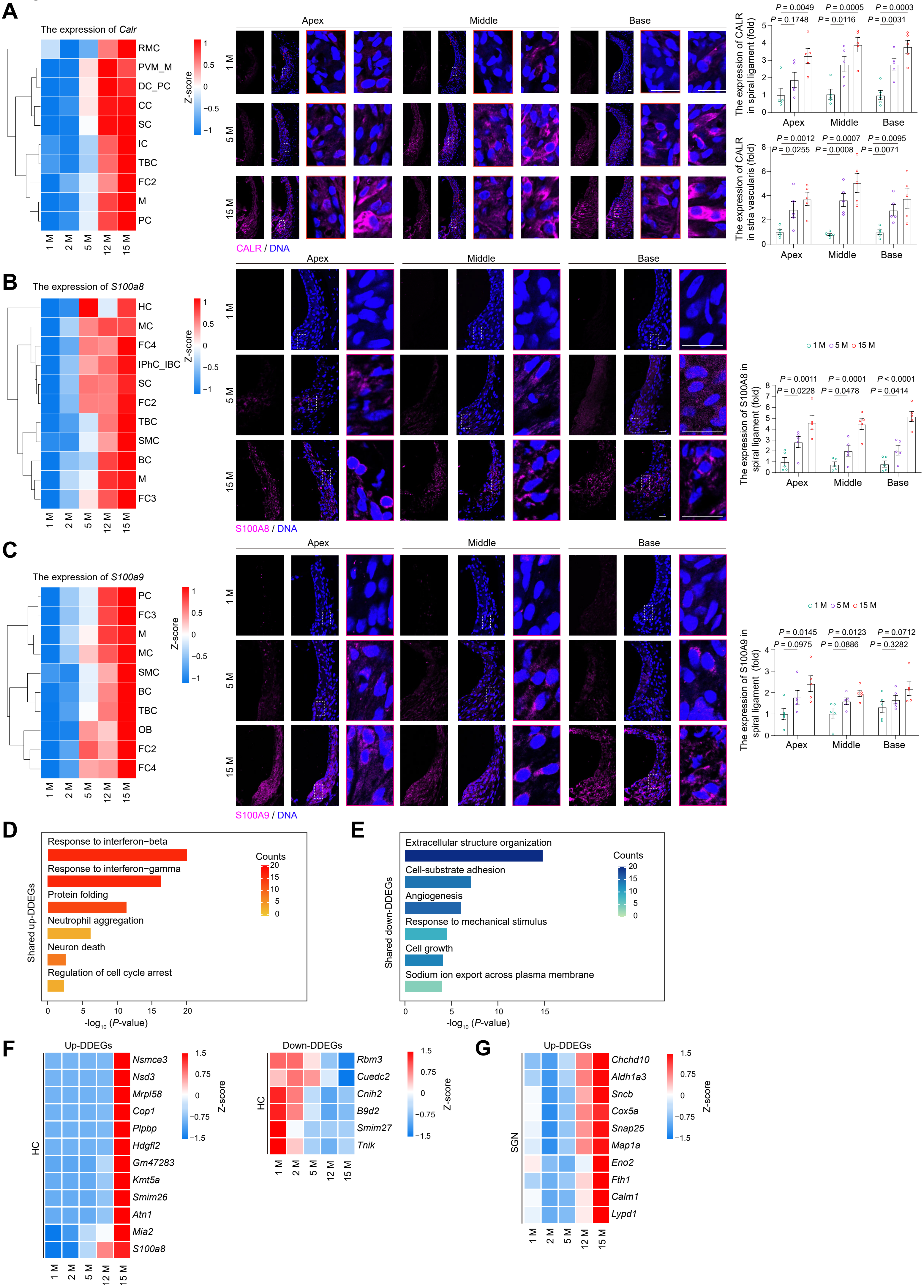
A
The expression of Calr
RMC
PVM_M
DC_PC
CC
SC
IC
TBC
FC2
M
PC
1 M
2 M
5 M
12 M
15 M
Z-score
1
0.5
0
−0.5
−1
Apex
Middle
Base
CALR / DNA
1 M 5 M 15 M
P = 0.0049 P = 0.0005 P = 0.0003
P = 0.1748 P = 0.0116 P = 0.0031
The expression of CALR in spiral ligament (fold)
P = 0.0012 P = 0.0007 P = 0.0095
P = 0.0255 P = 0.0008 P = 0.0071
The expression of CALR in stria vascularis (fold)
B
The expression of S100a8
HC
MC
FC4
IPhC_IBC
SC
FC2
TBC
SMC
BC
M
FC3
S100A8 / DNA
P = 0.0011 P = 0.0001 P < 0.0001
P = 0.0228 P = 0.0478 P = 0.0414
The expression of S100A8 in spiral ligament (fold)
C
The expression of S100a9
PC
FC3
M
MC
SMC
BC
TBC
OB
FC2
FC4
S100A9 / DNA
P = 0.0145 P = 0.0123 P = 0.0712
P = 0.0975 P = 0.0886 P = 0.3282
The expression of S100A9 in spiral ligament (fold)
D
Shared up-DDEGs
Response to interferon−beta
Response to interferon−gamma
Protein folding
Neutrophil aggregation
Neuron death
Regulation of cell cycle arrest
Counts
-log10 (P-value)
E
Shared down-DDEGs
Extracellular structure organization
Cell-substrate adhesion
Angiogenesis
Response to mechanical stimulus
Cell growth
Sodium ion export across plasma membrane
Counts
-log10 (P-value)
F
Up-DDEGs
HC
Nsmce3
Nsd3
Mrpl58
Cop1
Plpbp
Hdgfl2
Gm47283
Kmt5a
Smim26
Atn1
Mia2
S100a8
Down-DDEGs
Rbm3
Cuedc2
Cnih2
B9d2
Smim27
Tnik
Z-score
1.5
0.75
0
−0.75
−1.5
G
Up-DDEGs
SGN
Chchd10
Aldh1a3
Sncb
Cox5a
Snap25
Map1a
Eno2
Fth1
Calm1
Lypd1

Figure S10

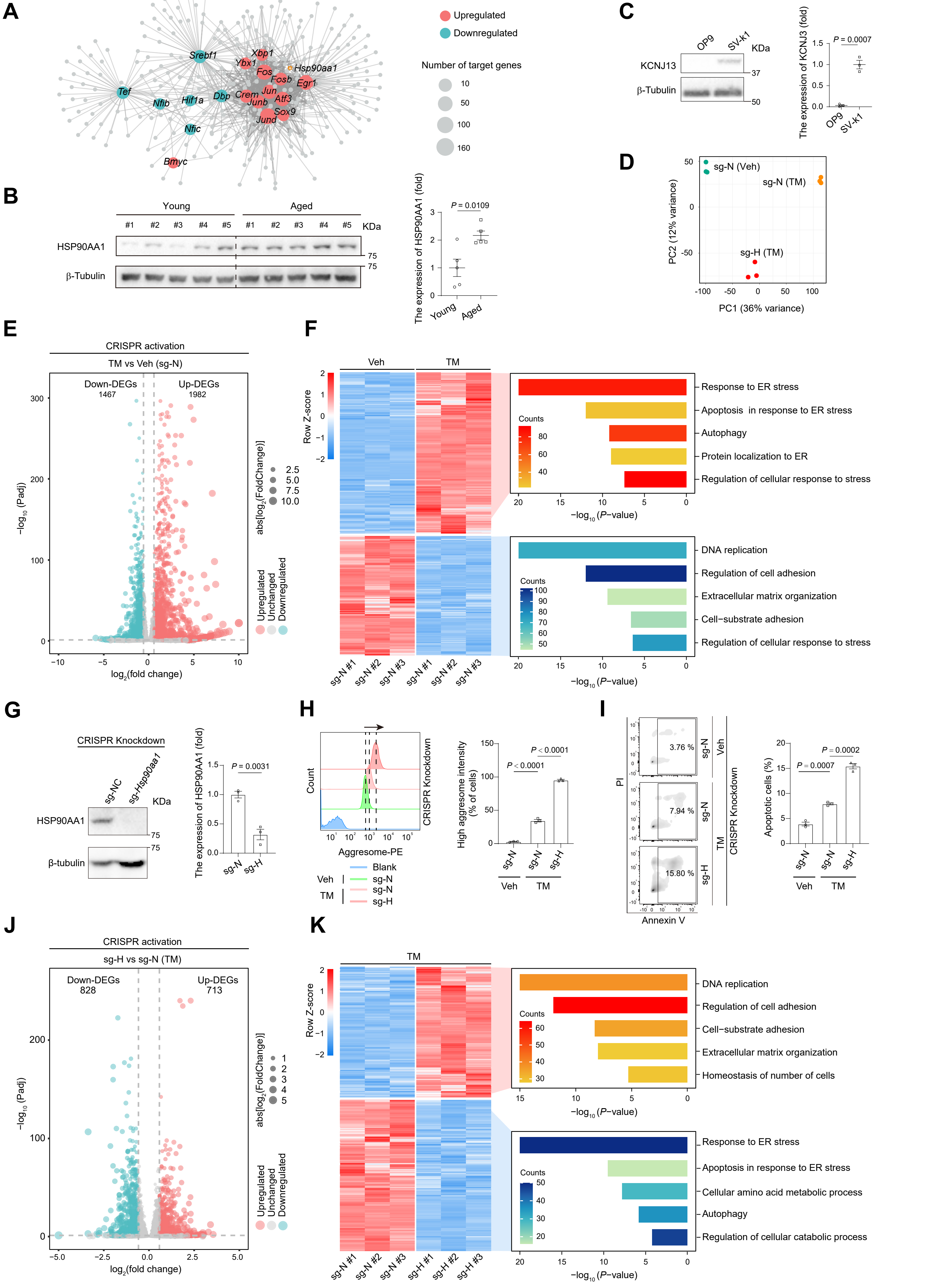

A
Upregulated
Downregulated
Number of target genes
10
50
100
160
B
Young
Aged
HSP90AA1
β-Tubulin
KDa
C
KCNJ13
β-Tubulin
OP9
SV-k1
D
sg-N (Veh)
sg-N (TM)
sg-H (TM)
PC1 (36% variance)
PC2 (12% variance)
E
CRISPR activation
TM vs Veh (sg-N)
Down-DEGs
1467
Up-DEGs
1982
log2(fold change)
−log10 (Padj)
F
Veh
TM
Row Z-score
Counts
Response to ER stress
Apoptosis in response to ER stress
Autophagy
Protein localization to ER
Regulation of cellular response to stress
DNA replication
Regulation of cell adhesion
Extracellular matrix organization
Cell−substrate adhesion
−log10 (P−value)
G
CRISPR Knockdown
sg-NC
sg-Hsp90aa1
HSP90AA1
β-tubulin
H
Count
Aggresome-PE
Blank
sg-N
sg-H
High aggresome intensity (% of cells)
I
PI
Annexin V
3.76 %
7.94 %
15.80 %
Apoptotic cells (%)
J
CRISPR activation
sg-H vs sg-N (TM)
Down-DEGs
828
Up-DEGs
713
K
TM
Homeostasis of number of cells
Cellular amino acid metabolic process
Regulation of cellular catabolic process
